# Therapeutic wavelengths of ultraviolet B radiation activate apoptotic, circadian rhythm, redox signalling and key canonical pathways in psoriatic epidermis

**DOI:** 10.1016/j.redox.2021.101924

**Published:** 2021-03-10

**Authors:** Rachel Addison, Sophie C. Weatherhead, Anandika Pawitri, Graham R. Smith, Ashley Rider, Henry J. Grantham, Simon J. Cockell, Nick J. Reynolds

**Affiliations:** a Institute of Translational and Clinical Medicine, Faculty of Medical Sciences, Framlington Place, Newcastle University, Newcastle Upon Tyne, UK; b Department of Dermatology, Royal Victoria Infirmary, Newcastle Hospitals NHS Foundation Trust, Newcastle Upon Tyne, UK; c Bioinformatics Support Unit, Faculty of Medical Sciences, Framlington Place, Newcastle University, Newcastle Upon Tyne, UK

**Keywords:** Transcriptomics, UVB phototherapy, Scalable biomarkers, Personalised therapy, p53 signalling, Epidermal remodelling

## Abstract

Ultraviolet B radiation (UVB) exerts pleiotropic effects on human skin. DNA damage response and repair pathways are activated by UVB; if damage cannot be repaired, apoptosis ensues. Although cumulative UVB exposure predisposes to skin cancer, UVB phototherapy is widely used as an effective treatment for psoriasis. Previous studies defined the therapeutic action spectrum of UVB and showed that psoriasis is resistant to apoptosis. This study aimed to investigate early molecular responses within psoriasis plaques following irradiation with single equi-erythemogenic doses of clinically-effective (311 nm, narrow-band) compared to clinically-ineffective (290 nm) UVB. Forty-eight micro-dissected epidermal samples from 20 psoriatic patients were analyzed using microarrays. Our bioinformatic analysis compared gene expression between 311 nm irradiated, 290 nm irradiated and control psoriasis epidermis to specifically identify 311 nm UVB differentially expressed genes (DEGs) and their upstream regulatory pathways. Key DEGs and pathways were validated by immunohistochemical analysis.

There was a dynamic induction and repression of 311 nm UVB DEGs between 6 h and 18 h, only a limited number of DEGs maintained their designated expression status between time-points. Key disease and function pathways included apoptosis, cell death, cell migration and leucocyte chemotaxis. DNA damage response pathways, NRF2-mediated oxidative stress response and P53 signalling were key nodes, interconnecting apoptosis and cell cycle arrest. Interferon signalling, dendritic cell maturation, granulocyte adhesion and atherosclerotic pathways were also differentially regulated. Consistent with these findings, top transcriptional regulators of 311 nm UVB DEGs related to: a) apoptosis, DNA damage response and cell cycle control; b) innate/acquired immune regulation and inflammation; c) hypoxia/redox response and angiogenesis; d) circadian rhythmicity; f) EGR/AP1 signalling and keratinocyte differentiation; and g) mitochondrial biogenesis.

This research provides important insights into the molecular targets of 311 nm UVB, underscoring key roles for apoptosis and cell death. These and the other key pathways delineated may be central to the therapeutic effects of 311 nm in psoriasis.

## Graphical abstract

Schematic representation of study to delineate pathways activated by therapeutic wavelengths of UVB in psoriasis.

**Figure:**
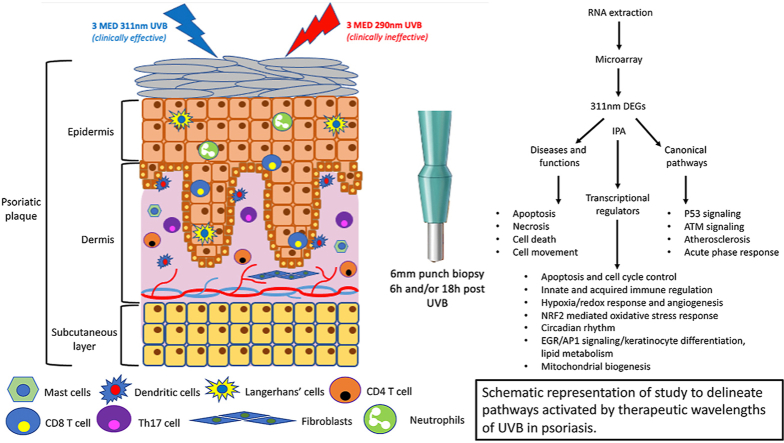

## Abbreviations

**AP-1**: Activator protein-1
**ATM**: Ataxia-telangiectasia mutatedse
**CRP**: C-reactive protein
**DASL**: cDNA-mediated annealing selection, extension and ligation
**DEGs**: Differentially expressed genes
**DUSP**: Dual specificity phosphatase
**EGR**: Early growth response
**IFN**: Interferon
**IPA**: Ingenuity Pathway Analysis
**IRF**: Interferon regulatory factor
**MED**: Minimal erythema dose
**MMP**: Matrix metalloproteinase
**nm**: nanometres
**OCT**: Optimal cutting temperature
**PCA**: Principal component analysis
**PCNA**: Proliferating cell nuclear antigen
**PDGF**: Platelet derived growth factor
**ROS**: Reactive oxygen species
**SCN**: Suprachiasmatic nucleus
**UVB**: Ultraviolet B radiation
**8-oxodG**: 8-oxo-2′-deoxyguanosine

## 1 Introduction

There is a wide variation in biological effects of ultraviolet radiation, dependent on wavelength. Ultraviolet B radiation (UVB, 290–320 nm), comprising approximately 6% of terrestrial summer solar radiation UV spectrum energy, induces a plethora of effects within human skin including DNA damage, reactive oxygen species (ROS) generation, apoptosis, cell cycle arrest, immunosuppression, cellular proliferation, vitamin D synthesis and cytokine release [1,2]. DNA is a chromophore within the epidermis, directly absorbing UVB which leads to formation of cyclobutane dimers and (6–4) photoproducts. In normal human skin, keratinocytes undergo apoptosis following UV irradiation at doses of around or greater than the minimal erythema dose (MED) [3,4] through a p53-dependent pathway.

Exposure to UVB irradiation may increase the level of ROS in the skin which can result in oxidative damage to proteins, nucleic acids and lipids [5]. ROS may be generated immediately through the direct effects of UVB on the cell membrane and cytoplasm or indirectly through later downstream effects on mitochondria. The timing and source of the ROS may exert differential effects on cellular processes [6]. Excessive production of ROS can disrupt redox homeostasis and contribute to DNA damage through the formation of cyclobutane pyrimidine dimers (CPDs), pyrimidine-pyrimidone (6–4) photoproducts and 8-oxo-2′-deoxyguanosine (8-oxodG) [7] and subsequently lead to apoptosis, inflammation and immunosuppression. p53 plays a crucial role in regulating gene transcription and modulating DNA repair in response to stressors such as UVB. Normal p53 function is tightly regulated by post-translational modifications and requires a reducing environment [8]. Overproduction of ROS, resulting in oxidative conditions, can alter the folding and function of p53 and thereby inhibit the repair of UV-induced DNA damage and/or inhibit apoptosis. This can result in an imbalance in genomic stability and permit malignant transformation within the cell cycle [7]. However, p53 also induces pro-oxidant and pro-apoptotic genes including PUMA, Bax and Noxa, suggesting complex inter-relationships and that p53 may influence cellular redox status.

For years it has been recognised that exposure to sunlight can improve psoriasis. Accordingly, UVB lamps have been developed which are highly effective in clearing psoriasis in 60–70% patients, [[9], [10], [11]]. Notably, UVB phototherapy may induce remission lasting for months after clearance has been achieved [[12], [13], [14]].

The action spectrum for clearance of psoriasis has been defined [15]. In a carefully conducted clinical study using repeated localised monochromatic irradiation to individual psoriasis plaques, Parrish and Jaenicke showed that wavelengths at or below 290 nm were ineffective – producing no clearance, even at 10–50 times the MED. On the other hand, wavelengths between 300 and 313 nm cleared psoriasis with 313 nm being the most effective. The mechanism of UVB-induced clearance of psoriasis has been the focus of numerous studies. However, previous work has looked for changes occurring following therapeutic UVB [[16], [17], [18], [19]]. Few previous studies have compared the early direct effects of therapeutic versus ineffective wavelengths of UVB, which would allow identification of important contributors to psoriasis clearance rather than bystander effects. We compared the early transcriptomic profile induced in lesional psoriasis skin *in vivo* by wavelengths of UVB that are effective (311 nm) to those that are ineffective (290 nm) at clearing psoriasis. By delivering equi-erythemogenic doses of 290 nm and 311 nm of UVB to matched sites, we controlled for processes not involved in the clearance of psoriasis, including those involved in generating erythema. We focused on the differential regulation of epidermal genes at 6 h and 18 h after a single irradiation of UVB (290 nm or 311 nm) (before cellular and clinical changes are detected), to identify important pathways in psoriasis clearance. Psoriasis has previously been shown to be relatively resistant to apoptosis *in vitro*, here we show here that clinically effective UVB *in vivo* induces apoptotic genes which later induce apoptosis of lesional basal/suprabasal keratinocytes [4,20].

## 2 Results

**Fig. 1:**
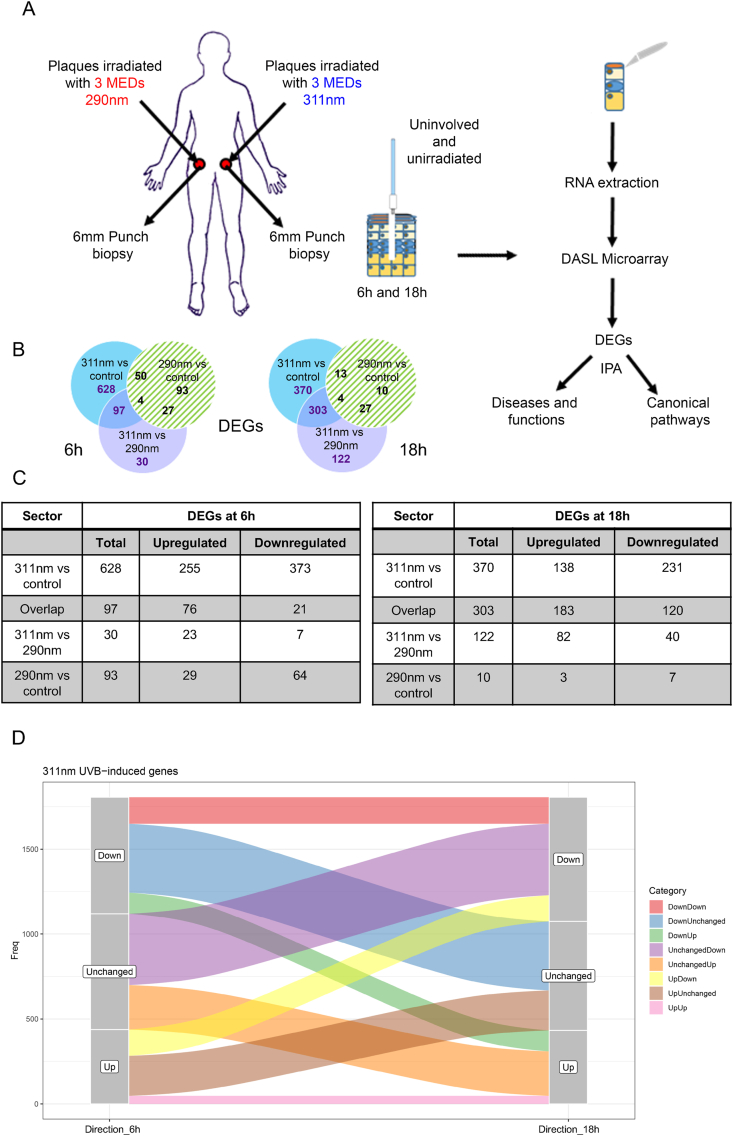
**Study Overview.** A) Schematic representation of irradiation protocol with either single exposure of 3 MEDs of 290 nm and/or 311 nm UVB to lesional psoriasis skin on the lower back. Six hours and/or 18 h later punch biopsies were taken from irradiated or unirradiated lesional psoriasis skin, microdissected, RNA extracted and subject to microarray and bioinformatic analysis. B) Venn diagrams showing DEGs derived from comparisons of 311 nm irradiated samples versus unirradiated controls, of 290 nm irradiated samples versus unirradiated controls, and 311 nm irradiated samples versus 290 nm irradiated samples at 6 h and 18 h. C) Numbers of upregulated and downregulated DEGs in each of the 3 key specified sectors (311 nm versus control, overlap and 311 nm versus 290 nm that comprise 311 nm UVB DEGs; excluding DEGs of 290 nm versus control) of the Venn Diagram. D) Alluvial plot showing the time course relationship of 311 nm UVB DEGs derived from the 3 key sectors (311 nm versus control, overlap and 311 nm versus 290 nm – excluding DEGs of 290 nm versus control) of the Venn diagram.

Fig. 1A provides a schematic representation of the study that involved 20 subjects with psoriasis who were referred for narrowband UVB phototherapy (311 nm UVB from herein). Prior to receiving phototherapy, subjects attended for localised irradiation of lesional (involved) psoriatic skin on the lower back, receiving a single 3 MED exposure of 311 nm UVB and/or 290 nm UVB. Skin biopsies were performed 6 h or 18 h after irradiation and/or from unirradiated psoriatic skin.

Transcriptomic analysis of RNA extracted from micro-dissected epidermis was performed. Principal component analysis (PCA) showed that relevant demographic or clinical variables did not significantly influence the overall variance of the transcriptomic data although sex was identified as contributing to PC2 (which represented 11.8% of the total variance) (Supplementary Fig. 1). Further analysis showed that sex was randomly assorted within the experimental groups and “sex specific” genes (genes differentially expressed by sex within the different experimental groups), and while present in the 311 nm response genes it accounted for <5% of the total at both time points (4.1% and 2.3% at 6 & 18 h respectively).

The numbers of differentially expressed genes (DEGs) induced by 311 nm UVB versus control, 311 nm UVB versus 290 nm UVB, and 290 nm UVB versus control are shown in the Venn diagram (Fig. 1B). As we were interested in genes specifically induced by therapeutically effective 311 nm UVB, we excluded from our analysis genes induced by 290 nm UVB compared to control (green hashed sectors). A summary of the most highly differentially regulated genes (within the blue and purple sectors of the Venn Diagrams; Fig. 1B; n = 755 at 6 h and n = 795 at 18 h), designated thereafter as 311 nm UVB DEGs, is given in Supplementary Table 1. The time-course relationship of the 311 nm UVB DEGs in the 3 key sectors is shown in Fig. 1D. This indicates that only a limited number of DEGs maintained their level of designated expression status (upregulated (n = 63) or downregulated (n = 42)) between 6 h and 18 h. Approximately equal numbers of upregulated and downregulated genes at 6 h became unchanged (compared to control) at 18 h. This was mirrored by an approximately equal number of unchanged genes (compared to control) at 6 h that became upregulated and downregulated at 18 h.

**Fig. 2:**
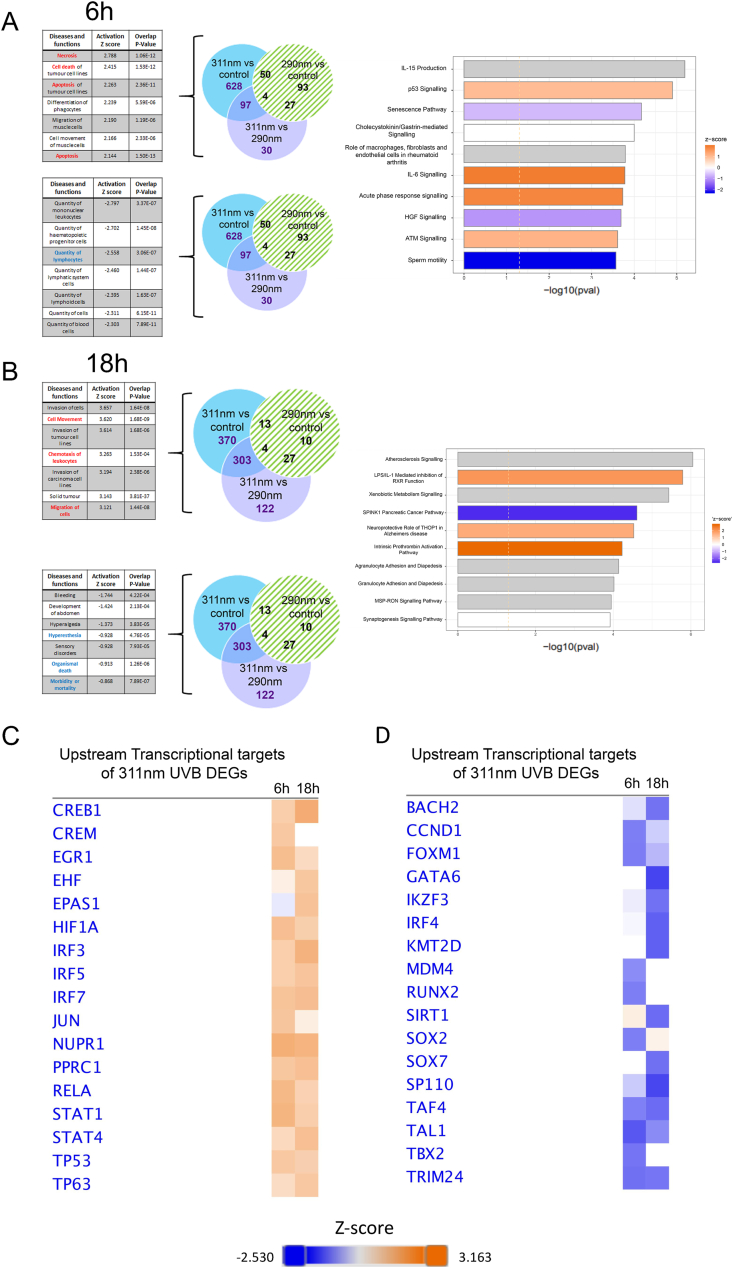
**Canonical pathways, diseases,functions and transcriptional regulators identified by IPA for DEGs in lesional psoriasis skin in response to 311 nm UVB compared to 290 nm UVB.** DEGs regulated by 311 nm UVB but not by 290 nm UVB (blue and purple sectors of Venn diagrams at 6 h (A) and 18 h (B) – 311 nm UVB DEGs) were analyzed by IPA. For diseases and function, the highest positive Z-scores and lowest negative Z-scores relating to the 311 nm DEGs (blue and purple sectors) are shown in upper and lower tables respectively for each time point. Canonical pathways differentially regulated by 311 nm compared to 290 nm (blue and purple sectors) are shown as horizontal bar graphs for each time point with Z-score represented by bar color. (C and D) Top transcriptional regulators differentially up-regulated (C) or down-regulated (D) by 311 nm at 6 h and 18 h are shown as heatmaps (C, darker orange represents greater expression and D) darker blue represents reduced expression at each time point). (For interpretation of the references to colour in this figure legend, the reader is referred to the Web version of this article.)

We used Ingenuity Pathway Analysis (IPA) on the 311 nm DEGs to understand the biological and functional significance of transcriptional changes induced by 311 nm UVB in lesional psoriasis skin and the inter-relationship of the DEGs at a systems level. Fig. 2A shows that a number of the upstream disease and function pathways related to induction of cell death or apoptosis at 6 h were significantly differentially positively regulated by 311 nm UVB, in line with effects of UVB in normal skin [21], and differentially increased apoptotic cells in psoriatic epidermis following 311 nm UVB compared to 290 nm UVB [22]. Interestingly, the quantities of several haematological-derived cell types, including lymphocytes and mononuclear leucocytes, were negatively regulated by 311 nm UVB at 6 h (Fig. 2A). At 18 h, the most differentially upregulated disease and function processes by 311 nm UVB related to cell movement, migration and chemotaxis. This was accompanied by significant differential regulation of the canonical pathways: granulocyte and agranulocyte adhesion and diapedesis (Fig. 2B). Genes of interest which are upregulated in these processes include FOSL1, IRF7, CXCR4, CXCL2, JUNB, GDF15, MMP1/3/7/9, MAPK3 and IL-6. Notably, metabolites from apoptotic cells have recently been shown to modulate gene expression programs in live cells with cell adhesion and migration being the largest module regulated [23].

### 2.1 Replication of array data

To replicate the findings of the array data, TaqMan® gene expression assays were performed for 5 genes - CDKN1A, DEF103A, FOSL1, GDF15 and KRT77 with a GAPDH loading control in 4 patients. These genes were chosen to represent differential regulation of genes at 6 h and 18 h post-irradiation following 311 nm, and differential regulation between 290 nm and 311 nm UVB.

These data demonstrated, with high concordance, that CDKN1A was upregulated 6 h post 311 nm UVB, FOSL1 was upregulated 6 h and 18 h post 311 nm UVB, and GDF15 was upregulated 6 h and 18 h post 311 nm UVB (Supplementary Fig. 2). Overall, these data correlated well with the gene array results.

**Fig. 3:**
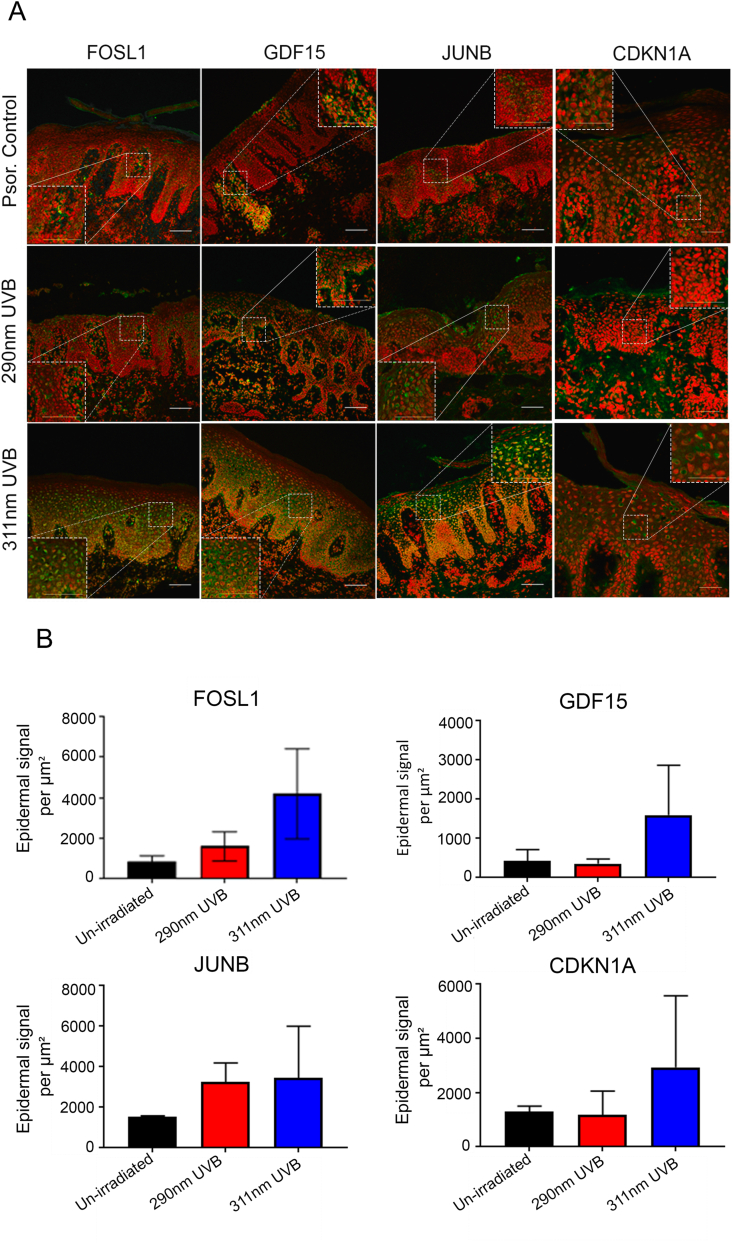
**311 nm UVB upregulates expression of nuclear FOSL1, GDF15, JUNB and CDKN1A in humanpsoriaticepidermis compared to 290 nmUVBand unirradiated control at 24 h.** (A) Confocal images of immunostained sections from biopsies taken 24 h after irradiation of lesional psoriasis skin (Psor.) with 3 MEDs of 311 nm UVB, 290 nm UVB or unirradiated control, captured using a scanning confocal microscopy (Leica TCP SP8). Scale bars for all images 100 μm , x40 oil immersion lens). All images shown were taken from the same donor. Red fluorescent signal – nuclear dye (TO-PRO) and green signal (Alexa Fluor 488 nm). (B) Volocity analysis was performed to quantify epidermal signal per μm^2^ (mean ± SD from 3 independent donors) for biomarkers FOSL1, GDF15, JUNB and CDKN1A in 311 nm UVB irradiated lesional skin, 290 nm UVB irradiated lesional skin and unirradiated control. (For interpretation of the references to colour in this figure legend, the reader is referred to the Web version of this article.)

To further validate findings from our transcriptional analysis, we performed immunohistochemical analysis focusing on selected apoptotic and cell cycle regulatory proteins (FOSL1, GDF15, JUNB and CDKN1A) (Fig. 3) in lesional psoriasis skin (control) and 24 h after 311 nm or 290 nm irradiation. Fig. 3A and B shows that CDKN1A was predominantly localised to the nucleus in suprabasal keratinocytes and was upregulated by 311 nm UVB compared to 290 nm UVB and unirradiated control at this timepoint. It was also upregulated at 6 h (Supplementary Fig. 2), suggesting a lag phase between mRNA upregulation and protein changes at 24 h, which coincides with the time course of 311 nm-induced apoptosis observed in psoriasis [22].

Increased nuclear staining intensity of FOSL1 and JUNB within keratinocytes was observed in lesional psoriasis skin irradiated with 311 nm UVB compared to 290 nm UVB and/or unirradiated lesional control skin (Fig. 3A and B). The number of FOSL1 and JUNB positively stained keratinocytes was also increased by 311 nm UVB compared to both 290 nm and lesional controls and localised predominantly in suprabasal keratinocytes (Fig. 3A). In line with these results, mRNA expression of both FOSL1 and JUNB was upregulated at 18 h post 311 nm compared to 290 nm and unirradiated controls, but not at the earlier timepoint of 6 h.

GDF15 expression was cytoplasmic, consistent with its known role as a secreted protein. We found elevated intensity and expression of GDF15 within the cytoplasm of psoriatic epidermis in response to 311 nm compared to 290 nm and lesional controls at 24 h (Fig. 3A and B). GDF15 mRNA expression was also upregulated at both 6 h and 18 h. Together our data indicate early and sustained modulation of GDF15 in response to 311 nm UVB.

### 2.2 Apoptosis and cell cycle regulatory genes

Because we had previously observed differential induction of apoptotic cells in lesional psoriasis skin [22] by 311 nm UVB, we identified, by IPA, which of the 311 nm UVB DEGs in lesional psoriatic skin were related to apoptosis (n = 215/755 genes at 6 h and n = 198/795 at 18 h). Expression heatmaps demonstrate modulation of genes related to apoptosis predominantly by 311 nm UVB, but no clear relationship between demographic or relevant clinical factors and gene expression (Supplementary Fig. 3A and B).

**Fig. 4:**
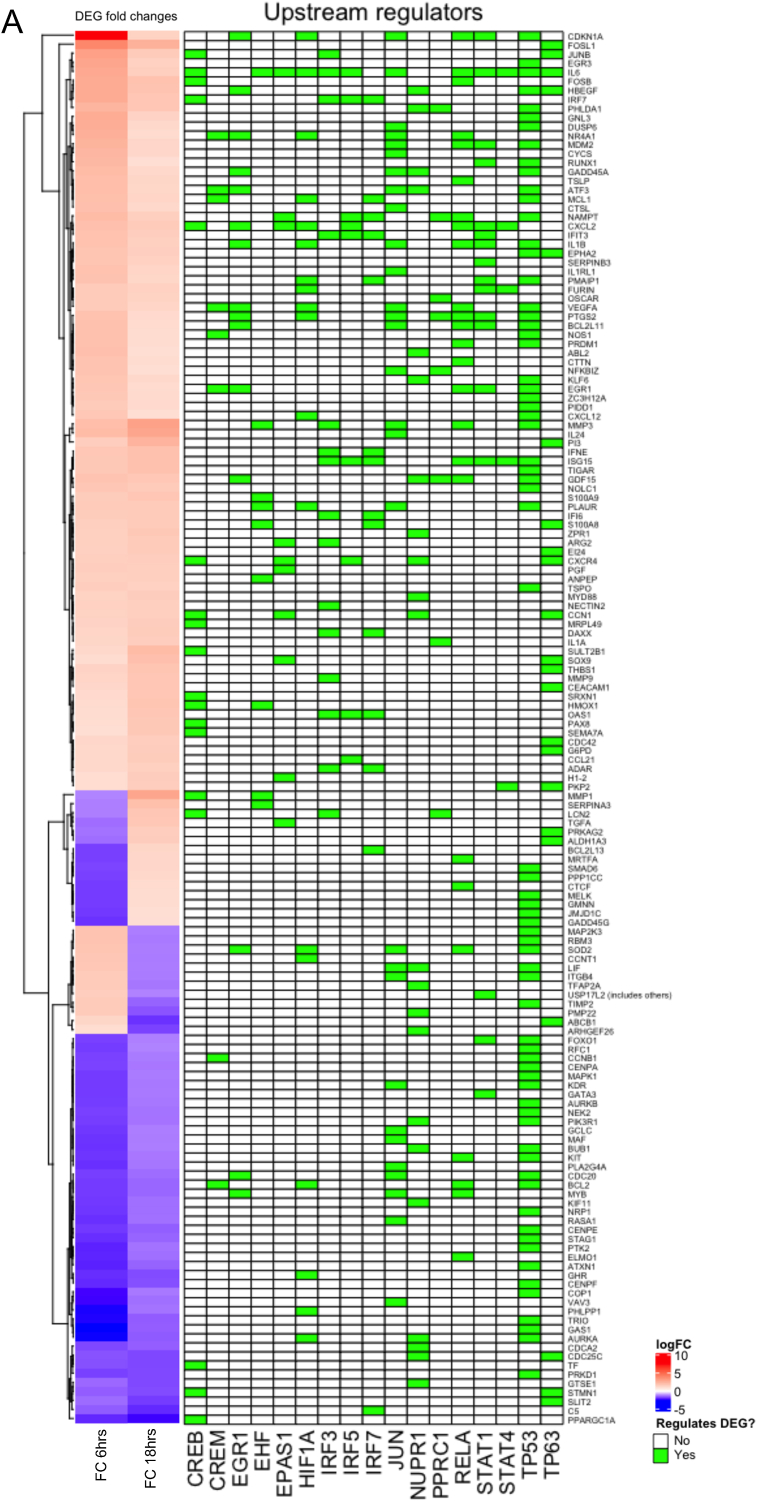

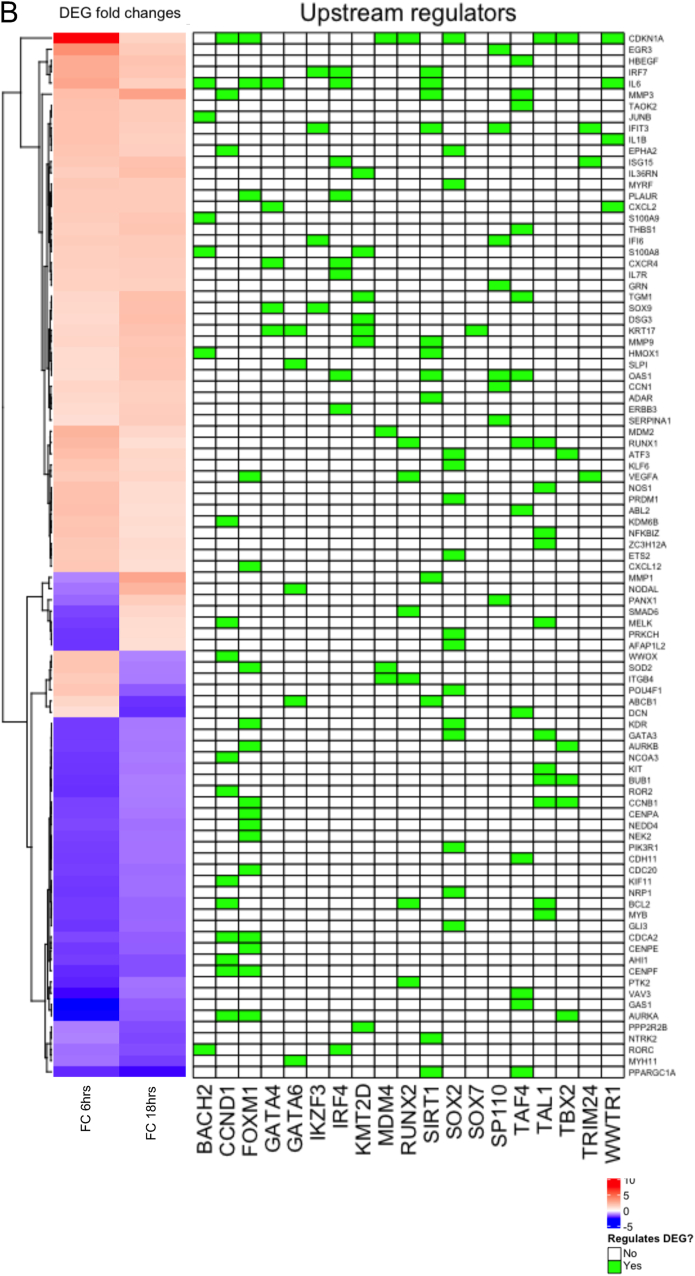
**Identification and visualisation of positive (A) and negative (B) upstream regulators of differentially expressed genes in lesional psoriasis skin induced by 311 nm UVB compared to 290 nm UVB at 6 h and 18 h after irradiation.** Heat maps show standardised gene expression (logFC) as; red: increased expression; blue: decreased expression. Gene lists on the right of the heatmap show apoptotic genes differentially regulated by the top upstream transcriptional regulators depicted across the bottom of the figure. Green boxes demonstrate which regulators regulate their respected DEGs. (For interpretation of the references to colour in this figure legend, the reader is referred to the Web version of this article.)

Pro-apoptotic genes such as IL-24, MMP3, GNL3, IL-36G, IFNE and GDF15 were differentially upregulated by 311 nm UVB at both 6 h and 18 h (Fig. 4A, Supplementary Fig. 4A and Supplementary Table 2). At the earlier timepoint, pro-apoptotic genes such as CALCA, COL4A and IL-17RD were upregulated and at the later time point, JUNB and FOSL1 were upregulated (Supplementary Fig. 3A and B). A range of anti-apoptotic genes, such as DACH1, GHR and NEK2 were downregulated at both time-points post UVB further sustaining a pro-apoptotic environment (Supplementary Table 2). In addition, a number of anti-apoptotic genes were downregulated at a single timepoint, such as BCL-2, TAF4 and AURKB 6 h post UVB and MYH11, RORC and SPOCK1 at 18 h (data not shown). Conversely, anti-apoptotic DEGs such as DYRK3, HAS3, MT1A and PPIF (6 h) and SERPINB4, ARF4, CCL21 and ARG2 (18 h) were upregulated at a single timepoint indicating some degree of heterogeneity in the regulation of anti-apoptotic genes (data not shown). Some anti-apoptotic DEGs were upregulated at both time points such as CXCL2, IFIT3, ISG15, SMOX, TIGAR and TREX contributing to an anti-apoptotic environment (Supplementary Table 2). Additionally, a number of pro-apoptotic DEGs were also key regulators of cell cycle progression; these include CDKN1A/p21, DUSP6 and MDM2 at 6 h post UVB. This may suggest a key role for these genes in early DNA damage response mechanisms which may be associated with the p53 signalling pathway (Fig. 4A). Altered expression of these DEGs is not sustained at the later time point.

Upstream regulators of 311 nm DEGs are discussed further below but notably these included EGR1, HIF1A, JUN, NUPR1, RELA and TP53 which are all classified as pro-apoptotic (Fig. 2C; Fig. 4A and B; Supplementary Table 3).

#### 2.2.1 p53 signalling pathway

p53 signalling preserves genomic stability by controlling a variety of DNA damage response machinery induced for example by UVB irradiation. Cell cycle arrest facilitates genomic repairs, but p53 signalling results in apoptosis when repair is not possible, preventing further replication and proliferation of transformed cells. However, prolonged exposure to UVB-irradiation can lead to the excessive production of ROS within the skin and inhibit p53 modulated DNA repair [7].

**Fig. 5:**
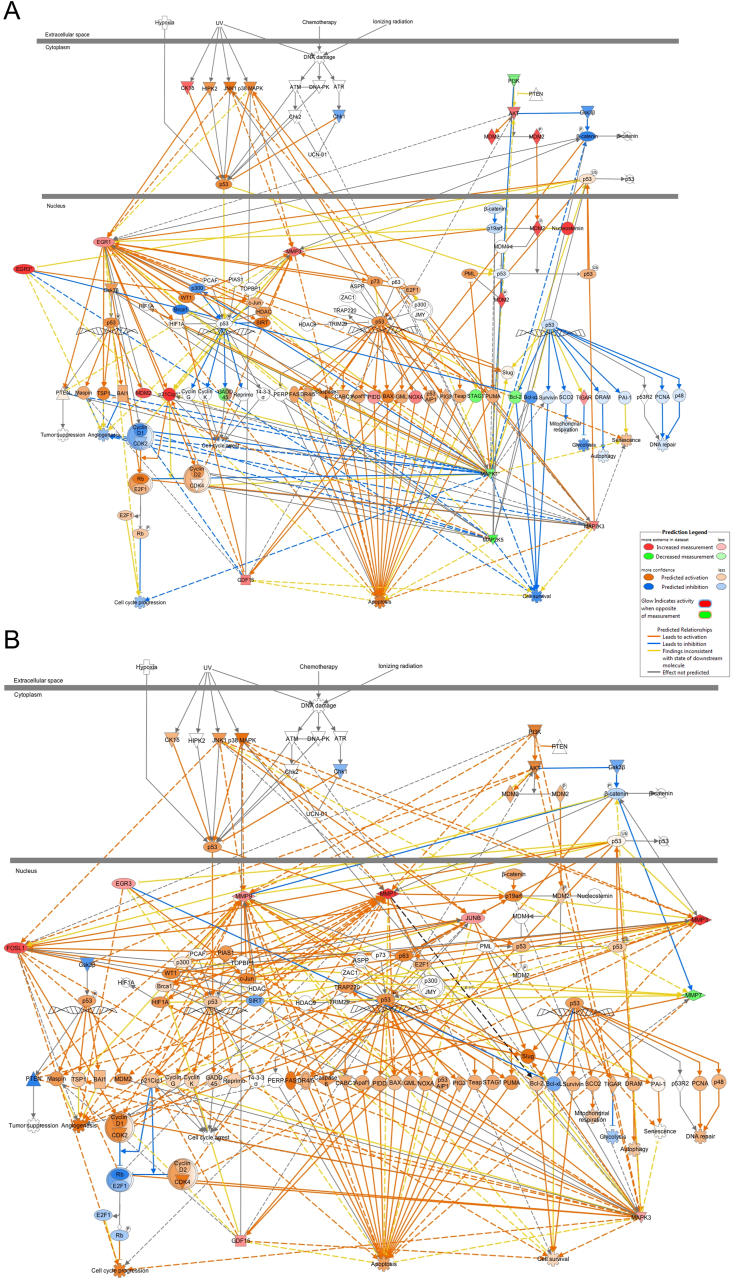
**Enriched canonical pathways of the DEGs associated with the p53 signalling pathway identified by IPA, showing gene expression and predicted relationships between top regulated 311 nm UVB DEGs at 6 h and 18 h post-irradiation.** Signalling pathway regulation based on the analysis of (A) 755 DEGs at 6 h and (B) 795 DEGs at 18 h respectively. Blue lines between DEGs represent predicted inhibition between genes and orange lines represent predicted activation between DEGs based on our transcriptomic data. Red symbols denote increased gene expression and green gene symbols represent downregulation. Each symbol shape represents a different molecule type. (For interpretation of the references to colour in this figure legend, the reader is referred to the Web version of this article.)

In normal human skin, p53 pathway activation by UVB directly correlates with the level of DNA damage [24,25] but may also involve p38 MAPK (MAPK14) and JNK1 signalling [26,27] (Fig. 5A and B). IPA demonstrated a central role for p53 in 311 nm-induced apoptosis in psoriasis with a large proportion of DEGs being associated with this pathway (Figs. 4A and 5A). p53 protein and RNA are upregulated in lesional psoriatic skin [28] compared to non-lesional skin. We did not observe further significant induction of TP53 mRNA in lesional psoriasis at 6 h or 18 h by 311 nm UVB. However, p53 protein was predicted to be upregulated at 6 h after 311 nm UVB irradiation (Fig. 5A and Supplementary Fig. 4) by IPA and we observed upregulation of p53 nuclear protein at 18 h by 311 nm but not 290 nm UVB in lesional psoriasis skin (Supplementary Fig. 5).

Fig. 5 highlights 311 nm DEGs upregulated in our dataset (red symbols) by p53 that promote apoptosis through the intrinsic (mitochondrial) pathway (e.g. NOXA) and downregulated (green symbols) anti-apoptotic genes such as BCL-2. As BCL-2 is overexpressed in psoriatic epidermis compared to normal epidermis [[29], [30], [31]], downregulation of BCL-2 by 311 nm UVB may contribute to increased apoptosis.

We identified differential expression of EGR1, MAP2K3 and CDKN1A at 6 h, FOSL1 and JUNB at 18 h, and EGR3 and GDF15 at 6 h and 18 h as key nodes within the p53 signalling pathway induced by 311 nm UVB in psoriasis (Figs. 4A and 5A and B and Supplementary Fig. 4A). UV is known to activate p38/MAPK in normal skin/keratinocytes [27] and promote the translocation of JNK to the nucleus where it phosphorylates and transactivates activator protein-1 (AP-1) complex members [32]. We found that this pathway was similarly activated in lesional psoriasis by 311 nm UVB (Fig. 5A and B). Interestingly, activation of p38/MAPK was also linked to upregulation of GDF15 and increased expression of pro-apoptotic intrinsic (mitochondrial) genes such as Bax and PUMA (orange symbols in Fig. 5A (predicted fold change)) [33] and extrinsic (initiated by death receptors) genes: TRAIL, FASL and TNF-α [34,35] (Fig. 5A and B).

Mitogen-activate protein kinase 1 (MAPK1) and MAP2K5 were both downregulated at 6 h following 311 nm UVB (Fig. 4A; Fig. 5 and Supplementary Fig. 4A). MAPK1 and MAPK3 both play an integral role within the MAPK/ERK signalling cascade that regulates keratinocyte proliferation, differentiation and cytokine production [36] (Fig. 4A and B; Supplementary Fig. 4 A and B). Activation of this signalling pathway is important in order to attenuate UVB-induced apoptosis. Downregulation of MAPK1 and MAP2K5 was predicted to negatively regulate cell survival at 6 h (Fig. 5A). At both time-points, UVB-induced modulation of p38/MAPK signalling resulted in upregulation of MAPK3. At 6 h, upregulation of MAPK negatively regulated cell survival and promoted apoptosis and was also predicted to be a key driver of p53 activation (Fig. 5A and B). However, at 18 h, upregulation of MAPK3 increased cell survival and promoted apoptosis to a lesser degree compared to the earlier time-point (Supplementary Table 1). This may suggest that p38/MAPK signalling may exert varying downstream effects dependent on time [37]. These data are consistent with a study in HaCaT keratinocytes which used key caspase inhibitors to block UVB-induced apoptosis [38]. This study demonstrated that UVB-induced activation of p38/MAPK resulted in apoptosis by inducing the release of mitochondrial cytochrome *c* into the cytosol followed by pro-caspase-3 cleavage [38].

GDF15 (growth differentiation factor 15; also known as MIC-1 or macrophage inhibitory cytokine-1), a member of the Transforming Growth Factor beta (TGF-β) superfamily, was differentially upregulated at both 6 h and 18 h by 311 nm UVB (Fig. 4A; Supplementary Fig. 4; Supplementary Table 1). Several upstream regulators of GDF15 were identified including PPRC1, NUPR1, EGR1 and EGR3. GDF15 is a transcriptional target for p53 and induces apoptosis [39]. It also plays a key role in regulating Cyclin D1(CCND1)/CDK2 interactions with E2F1/Rb which are key regulators of the cell cycle [40]. Interestingly, at 6 h GDF15 was predicted to negatively regulate Cyclin D1/CDK2 and upregulate E2F1/Rb subsequently inhibiting cell cycle progression. At 18 h, GDF15 was predicted to positively regulate Cyclin D1/CDK2 resulting in downregulation of E2F1/Rb, suggesting either adequate DNA damage responses permit safe cell cycle re-entry or apoptosis is required (Fig. 5B).

Matrix metalloproteinase (MMP) 1, MMP3 and MMP9 are largely expressed in basal keratinocytes and are key p53-regulated enzymes playing a central role in apoptosis by interrupting extracellular matrix survival signalling [41,42]. All three genes were upregulated by 311 nm UVB at 18 h with MMP3 also upregulated at 6 h (Fig. 5A and B; Supplementary Table 1). This is consistent with upregulation of MMPs in keratinocytes irradiated with low doses of UVB [43]. Upregulation of MMPs has been demonstrated with other lifestyle behaviours and disease states that lead to increased ROS production, such as smoking and cancer [44].

CDKN1A was the most significantly upregulated 311 nm UVB DEG at 6 h (Fig. 4A and B; Supplementary Fig. 3A; Supplementary Table 1). Interestingly, CDKN1A mRNA expression remained elevated 18 h after 311 nm UVB and was accompanied by increased CDKN1A protein (p21) expression at 24 h (Fig. 3; Supplementary Fig. 2A and B) and upregulation of MDM2 (Fig. 4A, Supplementary Fig. 4A, Supplementary Table 1). Upregulation of CDKN1A by UVB is tightly controlled by MDM2 and p53 [45,46]. CDKN1A regulates cell cycle progression at G1 by inhibiting cyclin-CDK2 or -CDK4 complexes, regulates DNA repair in S-phase by interacting with proliferating cell nuclear antigen (PCNA), and has p53 independent effects on cell cycle and apoptosis [47] (Fig. 2A and C; Fig. 5A and B). CDK2 and CDK4 modulate cell cycle progression by inducing Rb-dependent repression of E2F-mediated transcription (Fig. 5A and B). Together these data provide evidence for a role of CDKN1A in 311 nm UVB-induced growth arrest in hyperproliferative psoriatic plaques.

To gain greater understanding at a systems level of the inter-relationship of 311 nm UVB DEGs, we focused on canonical pathways identified by IPA. Ataxia-telangiectasia mutated (ATM) signalling was one of the top upregulated canonical pathways at 6 h in our data (ranked according to Z-score), consistent with its role as a key regulator of early DNA damage responses (Fig. 2A). ATM is primarily activated in response to double-stranded DNA breaks [48] and maintains genomic DNA damage response machinery to the DNA damage lesion. This machinery determines cell fate including apoptosis. Double-strand breaks are detected by MRE11 which was upregulated 6 h post UVB irradiation (data not shown). This time-course is consistent with an immediate response to UVB-induced DNA damage being mediated through ATM and p53 signalling in lesional psoriasis, similar to normal skin [21,49,50]. In addition, DNA damage response pathways such as p53 signalling promote differentiation to expel transformed cells from ‘normal’ proliferating cells when DNA damage is irreparable.

Not all genes followed a predictable pattern. For example, STAG1, a pro-apoptotic regulator which plays a key role in sister chromatid cohesion at telomeres and centromeres [51] (Zhao, Lin et al., 2019), was significantly downregulated at 6 h but was upregulated 18 h post-irradiation. This may suggest a lag-response of pro-apoptotic functions of STAG1 to UVB irradiation.

Within our data, senescence signalling was also downregulated 6 h post 311 nm UVB. DEGs within this canonical pathway include; MDM2, CDKN1A, IL-6, MRE11 and MAP2K3, which were all upregulated. Research has demonstrated that keratinocytes cultured *in vitro* resist UV-induced apoptosis whilst undergoing spontaneous replicative senescence and thus possess low levels of p53 [4]. Our data demonstrates upregulation of p53 signalling and downregulation of senescence signalling supporting the notion that keratinocytes within lesional skin are susceptible to UVB-induced apoptosis in response to a therapeutic wavelength of UVB (311 nm).

Early response genes induced by p53 that regulate cellular redox homeostasis were identified within our 311 nm UVB DEGs and included TIGAR (TP53-inducible glycolysis and apoptosis regulator) and CDKN1A protein. TIGAR, which was upregulated at both 6 h and 18 h (1.7 fold change and 1.9 fold change respectively), lowers fructose-2,6-bisphosphate levels, inhibiting glycolysis and thereby reducing overall cellular ROS [8] (Fig. 5A and B). Under low levels of stress, TIGAR forms part of the cells p53-dependent DNA damage response, indirectly inducing cell arrest by depleting cellular ATP. Interestingly, as disruption of metabolic homeostasis has been implicated in psoriasis pathogenesis, downregulation of glycolysis by 311 nm UVB may be relevant to the therapeutic effect of UVB phototherapy [52]. Additionally, CDKN1A protein, induced by p53, stabilizes the master antioxidant regulator NRF2, with the potential for further feedback loops as NRF2 itself induces CDKN1A, SESN2 and MDM2 [8]. Notably, the canonical pathway ‘NRF2 mediated oxidative stress response’ was significantly enhanced (P < 0.005) amongst 311 nm DEGs (Supplementary Fig. 6) and SESN2 was upregulated by 311 nm UVB vs control at 6 h and 18 h.

Further research is required to confirm these findings at the protein level and to elucidate the relevance of ROS signalling mechanisms in mediating the effects of 311 nm UVB in psoriasis.

#### 2.2.2 AP-1 signalling pathway and keratinocyte differentiation

A number of the key nodes in the p53 signalling pathway were AP-1 transcription factors, that comprise members of the FOS and JUN family which dimerize via a leucine zipper motif. 311 nm UVB DEGs included FOSB at 6 h and 18 h and also FOSL1 (FRA1) and JUNB at 18 h (Fig. 4A and B; Fig. 5B; Supplementary Table 1) which are highly expressed in psoriasis [53]. AP-1 transcription factors may play different roles in regulating differentiation, apoptosis and proliferation based upon epidermal location i.e. suprabasal and basal expression [[54], [55], [56]]. Furthermore, UV-induced AP-1 DNA binding and activation is dependent on cellular redox status [57]. For example, upon reduction, a ‘redox switch’ involving an inter-molecular disulfide bond within the bZIP domain of FosB regulates a conformational change and allows DNA binding [58]. Conversely when oxidised, the disulfide bond prevents this conformation change thereby preventing binding of FosB heterodimers to DNA (Fig. 3). Thus, modification of one AP-1 transcription factor may subsequently affect the function of another, which may influence the overall biological outcome [59]. ERK1/2, JUN, and FOSL1 may also regulate NRF-mediated oxidative stress signalling responses (Supplementary Fig. 6).

FOSL1 expression is detectable in all layers of the epidermis apart from the basal compartment, whereas JUNB expression is detectable in all epidermal layers in normal skin [54,60]. JUNB can interact with STAT3 (upregulated in our data at 18 h). Downregulation of AP-1 transcription factors can lead to increased proliferation and delayed keratinocyte differentiation [59]. This has been demonstrated in mouse models whereby conditional deletion of JUN expression in the epidermis led to altered keratinocyte differentiation and enhanced proliferation resulting in a psoriatic-like phenotype [61], dependent in part on enhanced TNFα production and loss of IL-6 suppression [53]. Thus, upregulation of JUN by 311 nm UVB would be predicted to reduce epidermal proliferation and enhance keratinocyte differentiation. In our IPA, JUN was ranked as a top transcriptional regulator at 6 h post 311 nm UVB (Fig. 2C; Supplementary Table 3) and FOSL1 and JUNB were upregulated 18 h post UVB phototherapy. This suggests UVB may have different effects on a number of AP-1 complex regulated DEGs associated with keratinocyte differentiation, such as IL-6 [62].

Immunohistochemistry of AP-1 transcription factors (FOSL1 and JUNB) (Fig. 3A and B) showed markedly increased suprabasal expression in response to 311 nm UVB in lesional psoriatic skin. Further, published single-cell RNA-seq data suggests a complex network of transcription factors and feedback mechanisms may drive proliferation, differentiation and apoptosis in AP-1 complex components [63]. Finnegan et al. demonstrated peak expression of both FOSL1 and JUN transcription factors (in normal skin) in early stage basal keratinocytes, with the lowest expression observed in mitotic keratinocytes. Specifically, both these transcription factors may act as commitment genes and serve as key drivers of keratinocyte differentiation.

CTSL plays a key role in keratinocyte proliferation [64]. Basal keratinocytes deficient in this gene have been shown to undergo hyperproliferation [64]. In our data, CTSL was upregulated by JUN 6 h post UVB irradiation, suggesting an anti-proliferative effect (Fig. 4A). Other DEGs upregulated by JUN which are associated with keratinocyte differentiation include CDKN1A, IL-6, IL-24 and PTGS2 (Fig. 4A) [62,65].

DUSP (dual specificity phosphatase) 6 was differentially upregulated by JUN (transcription factor) at 6 h by 311 nm UVB (Fig. 4A; Supplementary Table 1). DUSP6 is expressed in granulocytes, monocytes and keratinocytes, negatively regulates the ERK/MAPK superfamily and positively regulates AP-1 transcription factors [66,67]. p53 may also target DUSP6, promoting cell death by modulating ERK phosphorylation and control of BCL-2 expression [68,69]. By regulating cell death and differentiation in both immune cells and epidermal keratinocytes, DUSP6 represents a credible therapeutic target for 311 nm UVB in psoriasis.

#### 2.2.3 T cell-mediated apoptosis

We previously showed that 311 nm UVB induces apoptosis of keratinocytes and T cells and is non-discriminatory in its cellular target [22]. Following 311 nm irradiation of psoriasis, IL-24 was upregulated at 6 h and 18 h but maximally at the later time-point (Fig. 4A; Supplementary Fig. 4A; Supplementary Table 1), consistent with previous observations in normal skin [70]. IL-24 (MDA7), a member of the IL-10 family of cytokines, is predominantly expressed in immune cells such as T and B lymphocytes, but is also secreted by keratinocytes, and dampens uncontrolled T cell responses [70]. IL-24 induces several pro-apoptotic DEGs such as GADD genes and down-regulates anti-apoptotic proteins such as BCL-2 [71,72]. In agreement with this, we observed upregulation of GADD45A expression at 6 h post 311 nm irradiation (2.2 fold) and downregulation of BCL-2 at 6 h post 311 nm UVB (Fig. 5A; Supplementary Table 1).

Further studies are required to localise cells overexpressing IL-24 and GADD45A in response to 311 nm UVB and to determine the functional upstream regulators of GADD45A in skin but we postulate that IL-24 signalling may contribute to plaque remodelling by suppressing keratinocyte proliferation, T cell responses and inducing apoptosis [73]. In support of this, GADD45 null mice showed reduced p38/JNK MAPK activation, p53 activity and apoptotic keratinocytes in response to UV irradiation [74].

### 2.3 Acute phase response

Acute phase response signalling is a key pathway activated in response to erythemogenic doses of UVB irradiation, mediating the infiltration of inflammatory cells such as dendritic cells and inflammatory cytokines [75]. Notably, acute phase response signalling was significantly upregulated in psoriasis at both 6 h and 18 h after 311 nm UVB (Z-score 1.807 and 3.162 respectively) (Fig. 2A). Genes regulated at both time-points included inflammatory cytokines such as IL-6, IL-36RN, IL-36G (expressed predominantly in the outer epidermis) [76] and NOLC1 which are associated with both acute phase response and pro-apoptotic signalling (Supplementary Table 1; Supplementary Table 2). Differential upregulation of acute phase response signalling in response to UVB in psoriasis is particularly interesting as lesional skin does not develop visible erythema even in response to doses many times in excess of the patient's MED [15], and, importantly, doses of 311 nm and 290 nm UVB were matched for their erythemogenic potential (in uninvolved skin). It has been demonstrated that clearance of psoriasis by phototherapy is associated with decreased acute inflammatory markers such as serum C-reactive protein (CRP) levels [77,78]. Given that biopsies were obtained from patients at 6 h and 18 h in this study, this is likely too early to show any UVB-induced anti-inflammatory effects (such as suppression of Th17 cells and some degree of suppression of certain Interferon (IFN) I and II signalling pathways) within lesional skin.

### 2.4 Atherosclerosis

Psoriasis is increasingly seen as a systemic immune mediated inflammatory disease associated with increased risk of co-morbidities such as atherosclerosis and diabetes mellitus type II [79,80]. Atherosclerosis results from abnormal signalling between immune mechanisms and metabolic processes within the vessel walls. Chronic inflammation, as seen for example in psoriasis, rheumatoid arthritis and atherosclerosis, may link mechanistically these disorders and lead to insulin resistance and subsequent endothelial dysfunction [[81], [82], [83], [84]].

Activated inflammatory cells and pro-inflammatory cytokines contribute and maintain psoriatic phenotype and may also negatively impact atherosclerotic lesion stability. Interestingly, atherosclerosis signalling was the top 311 nm differentially regulated canonical pathway identified by IPA (ranked according to p-value) at 18 h but the overall directionality of change was not predictable (Fig. 2B; Supplementary Fig. 7). In our dataset some pro-atherogenic genes were upregulated, such as IL-6 and MMP3, and others were downregulated, such as PDGF-D. Anti-atherogenic genes such as IL-1A were also upregulated. These findings may reflect the complexity of distinct cell type interactions and local versus distant effect of UVB on secreted cytokines and mediators.

Serum IL-6 levels have been shown to be significantly upregulated in both atherosclerotic and psoriatic plaques due to the elevated levels of inflammation in both diseases [85,86]. IL-6 was upregulated at both 6 h and 18 h post UVB irradiation though was more upregulated at the earlier time-point (2.7 and 1.5 fold respectively) (Fig. 4A and B; Supplementary Table 1). Interestingly, IL-6 can exert both pro- and anti-inflammatory roles in atherosclerosis [85]. IL-6 can induce vascular smooth muscle cell proliferation and macrophage lipid accumulation which together promote disease progression (Supplementary Fig. 7). However, IL-6 can also inhibit disease progression in macrophages by mediating upregulation of the lipid handling system via upregulation of ATP binding cassette transporter ABCA1, which is a protein involved in macrophage lipid efflux [85].

As well as its role in p53-mediated apoptosis, GDF15 is a stress-mediated inflammatory cytokine which can be expressed in cardiomyocytes, adipocytes, macrophages, endothelial cells and vascular smooth muscle cells in relation to atherosclerosis [87]. GDF15 levels increase in response to inflammatory conditions and this has been shown to contribute to endothelial dysfunction, vascular inflammation, metabolic syndrome and atherosclerosis pathogenesis [87,88]. Conversely, GDF15 may play a protective role in atherosclerosis by attenuating macrophage accumulation via the downregulation of adhesion molecules [89] and has also been identified as a good prognostic serum biomarker in chronic heart failure and myocardial infarction [90,91]. Specifically, GDF15-deficient mouse models demonstrate enhanced macrophage accumulation and signs of atherosclerotic plaque destabilisation [89]. Upregulation of GDF15 at both 6 h and 18 h in psoriasis plaques post 311 nm UVB may therefore represent activation of a signalling pathway that has the potential to contribute to psoriatic plaque resolution and anti-atherogenic activity.

Interestingly, 311 nm DEGs also included downregulation of platelet-derived growth factor (PDGF), which is a pro-atherogenic gene [92,93] (Supplementary Fig. 7). Our IPA demonstrated downregulation of PDGF and suggested subsequent downregulation of collagen I in smooth muscle cells. Consistent with this, PDGF plays an essential role in cell proliferation, cell migration, cell survival and increased rate of blood vessel maturation during angiogenesis [94,95]. PDGF also acts as a chemotactic factor for macrophages, neutrophils and fibroblasts. Downregulation of PDGF limits this recruitment and limits the migration of smooth muscle cells to affected blood vessels. PDGF is minimally expressed in normal epidermis; however, in psoriatic epidermis expression of PDGF is markedly elevated. Changes in PDGF and TGF-β expression have been hypothesised to contribute to psoriatic co-morbidities such as cardiovascular disease, atherosclerosis and fibrosis [96]. It is thus conceivable that the downregulation of PDGF at both 6 h and 18 h in psoriatic plaques post 311 nm UVB may represent dampening of a signalling pathway implicated in psoriasis and atherosclerosis pathogenesis. Consequently, such dampening has the potential to contribute to psoriatic plaque resolution and anti-atherogenic effects.

### 2.5 Upstream transcriptional regulators

We used IPA to identify upstream regulators which were predicted to be activated in response to 311 nm at either 6 h or 18 h post UVB (Fig. 2C and D; Supplementary Table 3). These regulators were ranked according to Z-score and the top associated 10 DEGs for each time-point were identified (Fig. 4A and B; Supplementary Fig. 4A and B).

Top transcriptional regulators of 311 nm UVB DEGs included those related to: a) apoptosis and cell cycle control (NUPR1, EGR1, EGR3, JUN, TP53, HIF1A); b) innate and acquired immune regulation and inflammation (STAT1, STAT4, RELA, IRF3, IRF7, IFIT3, CREB1); c) hypoxia/redox response and angiogenesis (HIF1A, EPAS1); d) synchronization of circadian rhythmicity (CREM, CREB1) (Supplementary Fig. 8A and B); e) lipid metabolism (NFKBIZ and SREBF1); f) EGR/AP1 signalling and keratinocyte differentiation (EGR1, EGR3, TP63, JUN, EHF); and g) mitochondrial biogenesis (PPRC1, which activates mitochondrial biogenesis, in part through a direct interaction with NRF1 in response to mitochondrial stress, and also induces GDF15 (upregulated in our data set) as part of the mitochondrial stress response) (Figs. 2C and 4A) [97]. Of these transcriptional regulators, only EGR1 and IRF7 were significantly differentially upregulated at both 6 h and 18 h following 311 nm UVB irradiation (Fig. 2C).

NUPR1 plays an important role in cell-cycle regulation, redox signalling, apoptosis, autophagy and DNA repair responses and known targets, including GDF15, MAGI1, HBEGF and AURKA (aurora kinase-A), were significantly differentially regulated by 311 nm UVB (Fig. 4A, Supplementary Fig. 4A). NUPR1 is localised to the nucleus of epidermal keratinocytes and transcriptionally regulates proteins involved in redox reactions, lipid metabolism and cell cycle regulation of keratinocytes in response to UVA [98]. AURKA, downregulated by 311 nm UVB at 6 h localises to the centrosome in early S-phase and regulates mitotic duplication, separation, spindle assembly and maturation. AURKA is elevated in untreated psoriatic epidermis compared to normal skin [99] and therefore downregulation of AURKA by UVB may contribute to cell cycle arrest in psoriatic lesions. AURKA may also be downregulated by TP53 (Fig. 4A; Supplementary Fig. 4A).

Early growth response (EGR) 1 and EGR3, C2H2-type zinc-finger proteins, were differentially up-regulated 6 h following irradiation with 311 nm UVB (Fig. 4A and B; Supplementary Table 1). EGR genes are positive regulators of extrinsic and intrinsic apoptosis, activating Fas ligands (FasL) [5,100] and transcriptionally regulate 311 nm DEGs such as CDKN1A and GDF15. In response to UVB, increased EGR expression in human skin is mediated via the activation of NF-κB [101]. Upregulation of EGR subsequently results in the regulation of key DNA damage response genes from the GADD45 (GADD45A is increased 2.2 fold in our data) family which promote cell cycle arrest, DNA damage responses and apoptosis [63,101] (Fig. 5A and B; Supplementary Table 1).

Conversely, inhibition of NF-κB and EGR expression suppresses UVB-induced cell-death [101]. Combined with our own findings, this suggest that a signalling cascade involving activation of NF-κB, EGR and GADD45 genes may facilitate UVB-induced cell death in psoriasis [101,102]. Additionally, GADD45 is a known circadian clock and p53 target gene and has the capacity to mediate JNK/p38MAPK activation and a ROS signalling loop [103]. This interaction can subsequently activate p53-regulated processes such as cell cycle arrest and apoptosis (Fig. 5A and B) [104,105] suggesting interconnection of a number of apoptotic pathways. Further studies at the protein level are indicated to confirm and further define the signalling and regulatory pathways activated in psoriasis plaques. EGR1 and 3 are highly expressed within the stratum granulosum and regulate late-stage epidermal differentiation [106]. EGR3 also plays a key role in T cell regulation and apoptosis [5,100]. Taken together, EGR proteins may play an important role in 311 nm-induced apoptosis and enhanced growth arrest/terminal differentiation in psoriasis and thereby contribute to plaque clearance.

HIF1A (hypoxia inducible factor 1 alpha) was significantly upregulated 6 h following irradiation with 311 nm UVB (Fig. 2C) and plays a key role in keratinocyte response to UVB irradiation [107]. HIF1A is a key transcriptional regulator of a number of genes necessary for cellular and systemic homeostatic responses in response to hypoxic conditions. HIF1A plays an essential role in energy metabolism, angiogenesis, differentiation and UVB-induced apoptosis in keratinocytes [[107], [108], [109]]. Angiogenesis is a key cellular process contributing to the pathogenesis and progression of chronic diseases such as psoriasis. mRNA and protein levels of HIF1A are elevated in psoriatic lesional keratinocytes compared to normal skin and correlate with IL-6 expression [110,111]. Moreover, increased HIF1A expression localises to the keratinocytes adjacent to the inflamed and elongated papillae in psoriasis. Together these data suggest important interactions between inflammatory cytokines such as IL-6 and HIF1A. UVB induces a HIF1A biphasic response through ROS generation in cultured primary keratinocytes. Immediate cytoplasmic ROS production results in downregulation of HIF1A expression whereas delayed mitochondrial ROS production results in upregulation of HIF1A expression [6]. The role of HIF1A in mediating the potential therapeutic effects of UVB in psoriasis warrants further study.

Type I IFN and downstream inducible genes play a key role in the early initiation of psoriatic lesions, following wounding, for example with the Koebner phenomenon [112]. Because type I IFNs may only be expressed transiently and may be difficult to detect, type I IFN-inducible genes are often utilised as a read-out for pathway activation. Repeated low-dose UVB irradiation of skin results in a type I IFN response [113]. Published data suggests that 311 nm UVB downregulates type I IFN signalling in line with lesion clearance over a period of 12 weeks [114]. Conversely, our study demonstrates a predominant upregulation of IFN signalling at 6 h and 18 h. This difference may be in part explained by an acute versus chronic response.

**Fig. 6:**
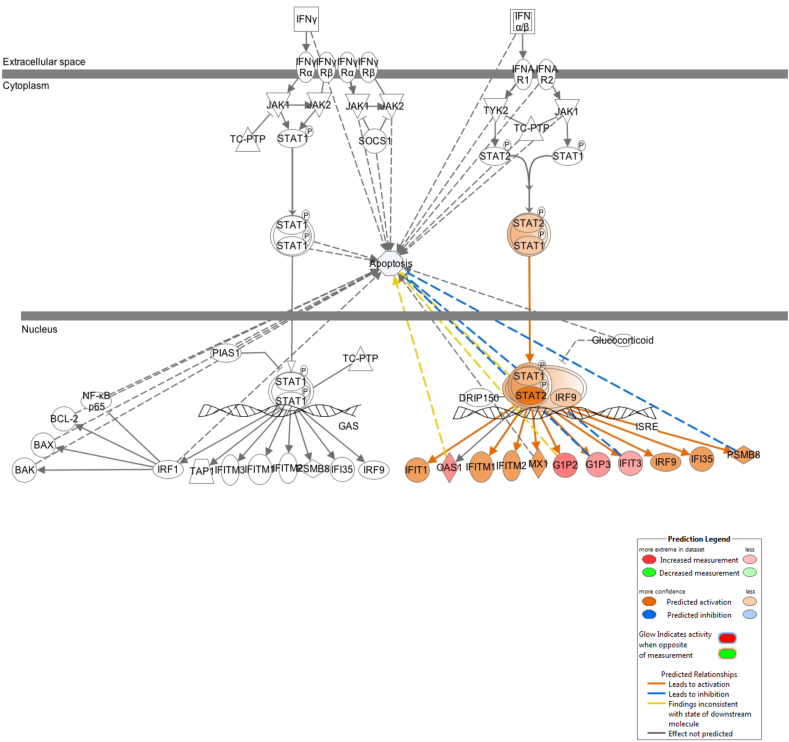
**Enriched canonical pathway of IFN transcriptomic pathway identified by IPA, showing gene expression and predicted relationships between molecules differentially regulated by 311 nm UVB 18 h after irradiation.** 311 nm UVB DEG expression and molecular relationships between 311 nm UVB DEGs predicted by using IPA molecular activity predictor using our transcriptomic data of 795 (18 h) DEGs. Transcriptomic data associated with apoptosis was applied to the IFN signalling pathway to identify any interconnectivity between IFN signalling and apoptosis. Blue lines between DEGs represent predicted inhibition between genes and orange represent predicted activation between DEGs based on our transcriptomic data. Red symbols denote increased gene expression. Each symbol shape represents a different molecule type. (For interpretation of the references to colour in this figure legend, the reader is referred to the Web version of this article.)

We found that several type I IFN-inducible genes, including interferon regulatory factor (IRF) 3, IRF5 and IRF7, were upregulated at both 6 h and 18 h, and that IFN pathway signalling was also upregulated 18 h post 311 nm UVB (Fig. 2C; Fig. 6). In addition to their role in regulating innate immunity and IFN signalling, a number of these transcription factors, including IRF1, 3, 6 and 7, have also been implicated in control of cell proliferation and keratinocyte differentiation [115]. IRF7 was differentially upregulated 6 h post 311 nm UVB (Fig. 2C), consistent with results in normal human keratinocytes and normal skin [116]; IRF7 was markedly induced in suprabasal keratinocytes by UVB. IRF7 is also expressed by plasmacytoid dendritic cells [117,118] and is overexpressed in psoriasis [119,120]. IRF6 is essential for normal epidermal development and differentiation and was upregulated by both 6 h and 18 h.

IFIT3 is an IFN-induced antiviral protein which inhibits viral processes, cell migration, proliferation, signalling and viral replication and was upregulated 18 h post 311 nm UVB [[121], [122], [123]]. IFIT3 mediates the phosphorylation of IRF3, subsequently resulting in the translocation of IRF3 to the nucleus in order to promote antiviral gene transcription. It also downregulates cellular proliferation by upregulating CDKN1A (p21 and p27) [122,124]. UVB-induced upregulation of IFIT3, IRF6 and IRF7 may contribute towards the decreased keratinocyte turn-over rate observed in lesional skin of patients treated with UVB phototherapy. IFIT3 also regulates innate immune responses and IFN gamma signalling and plays a key role in modulating apoptosis of keratinocytes (Fig. 6) [122,125].

Conversely, IRF4 was downregulated 18 h post 311 nm irradiation (Fig. 2D). Interestingly, in primary effusion lymphoma, treatment with immunomodulatory drugs also resulted in downregulation of IRF4 [126]. The transcription factor IRF4 plays a role in the development of naïve CD4^+^ and CD8^+^ T cell subsets, regulates a range of immune responses and can dictate T cell fate [127]. Unlike IRF3 and IRF7 (both key in antibacterial and antiviral immunity) and IRF5 (plays a pro-inflammatory and apoptotic role), IRF4 is restricted to lymphoid and myeloid lineages of the immune system and is crucial for B lymphocyte development and T helper cell differentiation [128]. In psoriasis, it is well documented that T cell differentiation is elevated in lesional skin. Myeloid-derived cytokines such as IL-12 and IL-23 also readily accumulate in affected skin [129]. The efficacy of drugs targeting IL-12/IL-23 demonstrate a key role for IL-23 signalling in psoriasis [130,131]. Downregulation of IRF4 by UVB in our study suggests that modulation of the IL-23/Th17 axis may be a key therapeutic target of UVB.

Genes uniquely regulated by IRF4 include CLTA, IL-7R, and PLAUR which are upregulated, and RORC and ZBTB20 which are downregulated 18 h post UVB (data not shown). Expression patterns of these genes all prove therapeutic in the clearance of psoriasis. To name but a few of these therapeutic effects; PLAUR is essential in influencing key pathological processes, these are driven by cell surface plasminogen activation [132]. This process drives degradation of ECM which is known to be key in resolving psoriatic plaques [133]; in mouse studies, RORC has been demonstrated to inhibit the expression of Fas ligands and IL-2. Fas ligand/receptor interactions are essential drivers of apoptosis and downregulation of RORC facilitates the presentation of Fas receptors and upregulates apoptosis [134].

Perhaps paradoxically, UVB-induced type I IFN proinflammatory cytokines may play a photo-protective role in attenuating inflammatory responses following repeated doses of UVB phototherapy [113]. Repeated doses of UVB irradiation in mouse models results in the induction of monocyte-dependent and plasmacytoid dendritic cell-independent expression of type I IFN and upregulation of other proinflammatory cytokines such as IL-1β, TNF and IL-6. In the context of psoriasis, UVB-induced type I inflammatory cytokines may contribute to the lack of increased erythema (and “burning”) observed in psoriatic plaques following UV or sun exposure.

Our IPA at 18 h following 311 nm UVB revealed activation of CREB1 and CREM. These act downstream of cAMP in the nucleus to regulate a wide variety of genes with cellular functions including proliferation, differentiation and survival, as well as adaptive immunity [135,136]. In psoriasis, cAMP signalling is reduced with CREB dysregulation, and this is thought to be involved in the development of new plaques [[137], [138], [139]]. Interestingly, cAMP, CREB and CREM have an essential role in the function of circadian rhythms, which are the daily variations in cellular activity found in all cells [140].

CREM regulates known circadian clock genes PERIOD, ARNTL (also known as BMAL1), EGR1 and NR4A1; CREB1 also regulates PERIOD and additionally HMOX1 and PPARGC1A (a master regulator and circadian coordinator of mitochondrial biogenesis and function) (Supplementary Fig. 8A and B and Supplementary Fig. 9). Notably, 23 out of 755 311 nm DEGs at 6 h overlapped with the 188 circadian rhythm genes delineated in skin [141] compared to 3 out of 188 290 nm DEGs (Supplementary Fig. 10A and C). At 18 h, 15/795 311 nm DEGs were classified as circadian clock genes whereas none of the 188 290 nm DEGs were (Supplementary Fig. 10B and D). Moreover, a high proportion of 311 nm circadian rhythm DEGs have key roles related to apoptosis (e.g CAMKK1, E2S2, GADD45A, THADA) and cell cycle control (e.g. AURKA, CDC20, IER5, KIF20A) (Supplementary Fig. 10A and B). CREB1 and CREM also have a role in the central circadian clock in the SCN in the brain whereby their activity is necessary to ensure correct alignment to environmental cues such as light, and cAMP is required for normal electrophysiological function [142,143]. In the skin, PERIOD3 reduces MMP1 activity (whose action is stimulated by UV, and other ROS-generating states, as mentioned above) through cAMP in a circadian fashion [144]. cAMP activation is also able to reduce UV-induced apoptosis by inhibiting JNK activity [145].

Lastly, regulation patterns of transcriptional regulators (ranked according to Z-scores) in response to 311 nm UVB demonstrated upregulation of a number of genes associated with lipid metabolism such as NFKBIZ and SREBF1. This may relate to the disruption of the epidermal barrier in psoriasis and potential of UVB to induce repair through modulation of keratinocyte lipid genes [146,147].

## 3 Concluding remarks

Utilising the defined action spectrum of clearance, and equi-erythemogenic doses of a clinically effective wavelength (311 nm) and a clinically ineffective wavelength (290 nm) of UVB, we were able to control for non-therapeutic effects (such as erythema induction) and thereby define early molecular events in psoriatic epidermis prior to clinical signs of resolution. Transcriptomic profiling combined with systems, upstream and protein analysis provides clear evidence that apoptosis and cell death pathways are activated in lesional psoriasis skin by therapeutic wavelengths of 311 nm UVB. Combined with our previous studies showing differential induction of apoptotic cells by 311 nm compared to 290 nm in lesional psoriasis [22], our findings support a key role for apoptosis in plaque clearance and epidermal remodelling. Consistent with our previous study [22], our analysis showed that lymphocytes and mononuclear leucocyte number and function were negatively regulated by 311 nm UVB at 6 h (Fig. 2A) and thereby likely contribute to psoriasis clearance. We found that DNA damage pathways and p53 signalling were key nodes (Figs. 4A and 5) interconnecting apoptosis and cell cycle arrest, and may be central to the therapeutic effects of 311 nm UVB in psoriasis. We also observed key regulation of ROS homeostatic DEGs (such as TIGAR) within the p53 signalling pathway in response to 311 nm at 6 h and 18 h. In the presence of mild/transient stress, TIGAR protects against ROS, induces growth arrest to allow DNA repair and modulates p53 apoptotic signalling. Under more severe/prolonged stress, p53 signalling results in apoptosis and in our analysis, the pro-apoptotic and pro-oxidant genes PUMA and Bax were both upregulated [148]. NRF2, previously shown to be induced by UVA, regulates oxidative stress response genes in keratinocytes [98]. Our data highlighting a NRF2-mediated oxidative stress response induced by 311 nm UVB (Supplementary Fig. 6) suggests a wider role for redox response in regulating epidermal homeostasis and response of psoriasis to UVB.

Several type I IFN-inducible genes were modulated by 311 nm UVB. Additional to their role in regulating innate immunity, IRF1, 3, 6 and 7, are expressed by keratinocytes and regulate differentiation [115]. Together with induction of AP1 members FOSB, FOSL1, JUN and JUNB, and EGR signalling, our data identify regulatory pathways induced by 311 nm UVB in psoriasis that control terminal differentiation of keratinocytes. Interestingly, we also found that circadian clock genes and pathways relating to psoriatic co-morbidities, including atherosclerosis, were modulated by 311 nm UVB.

By definition, bulk transcriptomic analysis of tissues does not distinguish the cell type from which the transcripts have been derived. Advances in the application of single cell RNA sequencing to skin samples are providing important insights into cell-type specific expression [149] and association of gene expression with cell type specific differentiation pathways. Combining single cell and bulk transcriptomic analysis of skin in future studies may result in increased understanding about the role of specific cell types in the response of normal and disease tissue to UVB and therapeutic agents.

Psoriasis is frequently associated with systemic long-term inflammatory conditions including metabolic syndrome and atherosclerosis, which is increasingly considered to reflect clustering of diseases with similar underlying risk factors and aetiology, including inflammasome activation [150]. Further, there is evidence of crosstalk between cutaneous psoriasis and these systemic conditions, such as activation of the IL23/type 17 T cell axis within psoriatic skin, which can influence metabolic syndrome progression [151,152]. Although clear differences exist between lesional and non-lesional psoriatic skin in terms of histology and inflammatory cell signature, non-lesional skin shows distinct differences compared to normal human skin including decreased lipid biosynthesis and increased innate immune activation [149,153,82]. Future studies should focus on integrating changes in key inflammatory and related pathways across tissues at a systems level and determine whether UVB phototherapy influences the development of associated co-morbidities in psoriasis.

## 4 Methods

### 4.1 Subjects

Twenty adult subjects (13 male and 7 female; mean age 48 years, range 18–69) with psoriasis were recruited from a tertiary referral dermatology clinic following the decision to treat their psoriasis with 311 nm UVB. Exclusion criteria included the use of sun-beds or sun exposure to the lower back for the preceding 3 months or use of topical anti-psoriatic treatments (with the exception of emollients) for 2 weeks, and the use of anti-psoriatic systemic therapies for 3 months, as per our previous protocol [22]. Following minimal erythema dose (MED) testing and irradiation of psoriatic skin with 290 nm and/or 311 nm UVB (section 4.2), skin biopsies were performed 6 h (± 2 h) and/or 18 h (± 1 h) later (section 4.3). Biopsies were also taken from unirradiated psoriatic skin (11 subjects) and unirradiated non-lesional skin (3 subjects). Patients then received routine clinical treatment with 311 nm (narrowband) UVB 3 times weekly. The initial starting dose was 0.7 MED and patients completed between 3 and 31 treatments (mean of 21.6 treatments). As part of a time course study, an additional 3 subjects were recruited. As above, lesional psoriasis skin was irradiated with a single 3 MED exposure of 311 nm and 290 nm UVB and skin biopsies taken 24 h later for immunochemical analysis (section 4.6).

This study had Regional Research Ethics Committee approval, and all subjects gave written informed consent to participate. The study was conducted according to the declaration of Helsinki Principles.

### 4.2 In vivo UVB irradiation

MED testing was performed on non-lesional (uninvolved) skin adjacent to the psoriatic plaques to be biopsied, using just perceptible erythema as a measure of 1 MED. The median MED was 459 J/cm^2^ (range 365–1158 J/cm^2^). Plaques were irradiated with 3 MEDs of 311 nm UVB from a handheld modified erythema test unit which had been allowed to warm up for 5 min to stabilise output (Hybec Ltd, Leicester, UK, fitted with a Phillips TL01 lamp; referred to as 311 nm in this paper) or 3 MED of 290 nm UVB using an irradiation monochromator set at a central wavelength of 290 nm (bandwidth 5 nm, measured as full width at half maximum height; MED range 6.3–31.7 J/cm^2^ median 12.6 J/cm ^2^), as per our previous protocol [22]. Involved (plaque) or uninvolved psoriatic skin (adjacent to an irradiated plaque) was irradiated in a maximum of four 15 mm diameter areas, in the sun-protected region of the lower back. Psoriatic plaques were chosen to be of similar size and thickness within each patient, and where possible small plaques were chosen which could be biopsied in full. For larger plaques, a biopsy was taken from the edge of a plaque.

### 4.3 Biopsies and RNA extraction

Six millimetre punch biopsies were taken from psoriatic plaques or adjacent non-lesional skin at 6 h (± 2 h) or 18 h (± 1 h) after irradiation with 311 nm or 290 nm UVB and from unirradiated psoriatic plaque skin. A maximum of four biopsies were taken per patient. Biopsies were snap frozen, set in OCT embedding medium (Raymond A Lamb) and stored at -80 °C. 35 μm cryosections were cut, collected on PEN slides (Leica, Milton Keynes) and kept frozen until microdissection. Slides were fixed in ethanol and stained with toluidine blue, and then the epidermis was micro-dissected with a scalpel using a dissection microscope. RNA was extracted using a Picopure TM RNA Isolation kit (Arcturus Biosciences, USA) using the manufacturers standard protocol, and stored at -80 °C.

### 4.4 Arrays

Samples were processed in 2 sets using cDNA-mediated Annealing, Selection, extension and Ligation (DASL) arrays (Illumina Inc, USA). Expression values were derived from importing raw data into GeneSpring GX 11.0 (Agilent technologies, USA). Expression values were log transformed (log base 2) and normalised using quantile normalisation with a baseline transformation to the median of all samples. Batch variability between the two sets of microarray data generated were removed using the empirical Bayes method implemented in ComBat, using the sva package [154] prior to analysis. Differential expression between groups was performed using the RankProducts package [155] in BioConductor [156]. Genes considered to be significantly different between groups were selected with a p-value of <0.05 after 100 permutations and a minimum 1.5 fold change. GeneSpring GX software allowed visualisation of the median and interquartile range for differentially regulated genes and further assesses the likely relevance of the observed fold changes. Further analysis was performed using Ingenuity Pathway Analysis 8.7 software and comparisons of gene expression were made within the following groups of psoriatic skin:

1. Untreated lesional vs untreated non-lesional
2. Untreated lesional vs 6 h post 311 nm irradiation
3. Untreated lesional vs 18 h post 311 nm irradiation
4. Untreated lesional vs 6 h post 290 nm irradiation
5. Untreated lesional vs 18 h post 290 nm irradiation
6. 6 h post irradiation with 311 nm vs 290 nm UVB
7. 18 h post irradiation with 311 nm vs 290 nm UVB
8. Irradiation with 311 nm after 6 h vs 18 h
9. Irradiation with 290 nm after 6 h vs 18 h

Heatmaps were generated in order to visualise gene expression patterns for DEGs associated with apoptosis at 6 h and 18 h post-irradiation with 311 nm, 290 nm UVB and unirradiated lesional control. Gene expression was Z-scored prior to input. All heatmaps were generated with the ComplexHeatmap package [157]. Apoptotic genes were clustered by an R pre-defined complete hierarchical clustering method.

### 4.5 Real-time PCR validation

Five genes were chosen to validate the array data in 4 patients. These were selected to represent significant differential regulation of genes following UVB-irradiation with 311 nm or 290 nm at either 6 h or 18 h compared to unirradiated psoriasis. TaqMan® gene expression assay probes (Applied Biosystems) were used for the following genes: CDKN1A, DEF103A, FOSL1, GDF15 and KRT77 with a GAPDH loading control. cDNA was prepared with a standard protocol using a Superscript™ III first-strand synthesis kit. Standard PCR cycle conditions were run for 40 cycles using a Chromo4 qPCR continuous fluorescence detector. Analysis was performed using the Delta-Delta Ct method after correction for the loading control [158].

### 4.6 Immunohistochemistry

Due to the complexity of our study design, and limitations on number of biopsies permitted per patient, this restricted the number of patients with biopsies obtained at 6 h and 18 h irradiated with 311 nm and 290 nm. For protein validation studies, we used biopsies obtained from 3 subjects at 24 h, snap frozen, and embedded in OCT. This also allowed us to account for any lag between mRNA and protein expression.

Cryosections of 6 μm thickness were fixed by using either 4% paraformaldehyde (0.4 g PFA in 10 ml PBS) or methanol and acetone (1:1), permeabilised with 0.2% triton X-100, blocked with normal goat serum (1:60) and incubated with specific monoclonal antibodies for biomarkers.

Four differentially expressed genes were chosen to validate in the epidermis of 3 representative patients. Monoclonal rabbit anti FOSL1 antibody (1:250, Abcam (ab124722)), monoclonal mouse anti JUNB (1:50 Santa Cruz (C-11)), monoclonal mouse anti GDF15 (1:1000, Abcam (ab106112)) and monoclonal mouse anti p21/CDKN1A (pre-diluted 4.15 μg/ml, Cell Marque) were used. Goat anti mouse or anti rabbit Alexa Fluor 488 (1:300, Invitrogen) were used as secondary antibodies. To-PRO (1:3000, Invitrogen) was used as a nuclear marker.

Primary antibodies and fluorochromes were diluted in 2% bovine serum albumin to their optimised dilution and incubated at room temperature.

Images of immunohistochemistry sections were captured using a Leica TCP SP8 scanning confocal microscope (Leica Microsystems, Germany) and a x40 oil immersion lens; 488 nm and 638 nm laser lines were used for imaging of Alexa Fluor 488 and To-PRO respectively. Epidermal fluorescent biomarker signal was quantified using Volocity® software (PerkinElmer, Inc, USA).

### 4.7 Data access

The array data has been deposited in the GEO repository - GEO Submission (GSE162998) [NCBI tracking system #21536878].

## Declaration of competing interest

NJR has received research grant funding from 10.13039/100004336Novartis, PSORT partners (www.PSORT.org.uk); and income to Newcastle University from Almirall, Leo, Lilly and Novartis for attendance at advisory boards.
