# Supplementary material for: Therapeutic wavelengths of ultraviolet B radiation activate apoptotic, circadian rhythm, redox signalling and key canonical pathways in psoriatic epidermis
Source: Redox Biol. 2021 Mar 10;41:101924. doi: 10.1016/j.redox.2021.101924 (PMC8050411; doi:10.1016/j.redox.2021.101924)
Supplement: Multimedia component 1 [file mmc1.pdf]

Supplementary figures/tables

Supplementary Figure 1

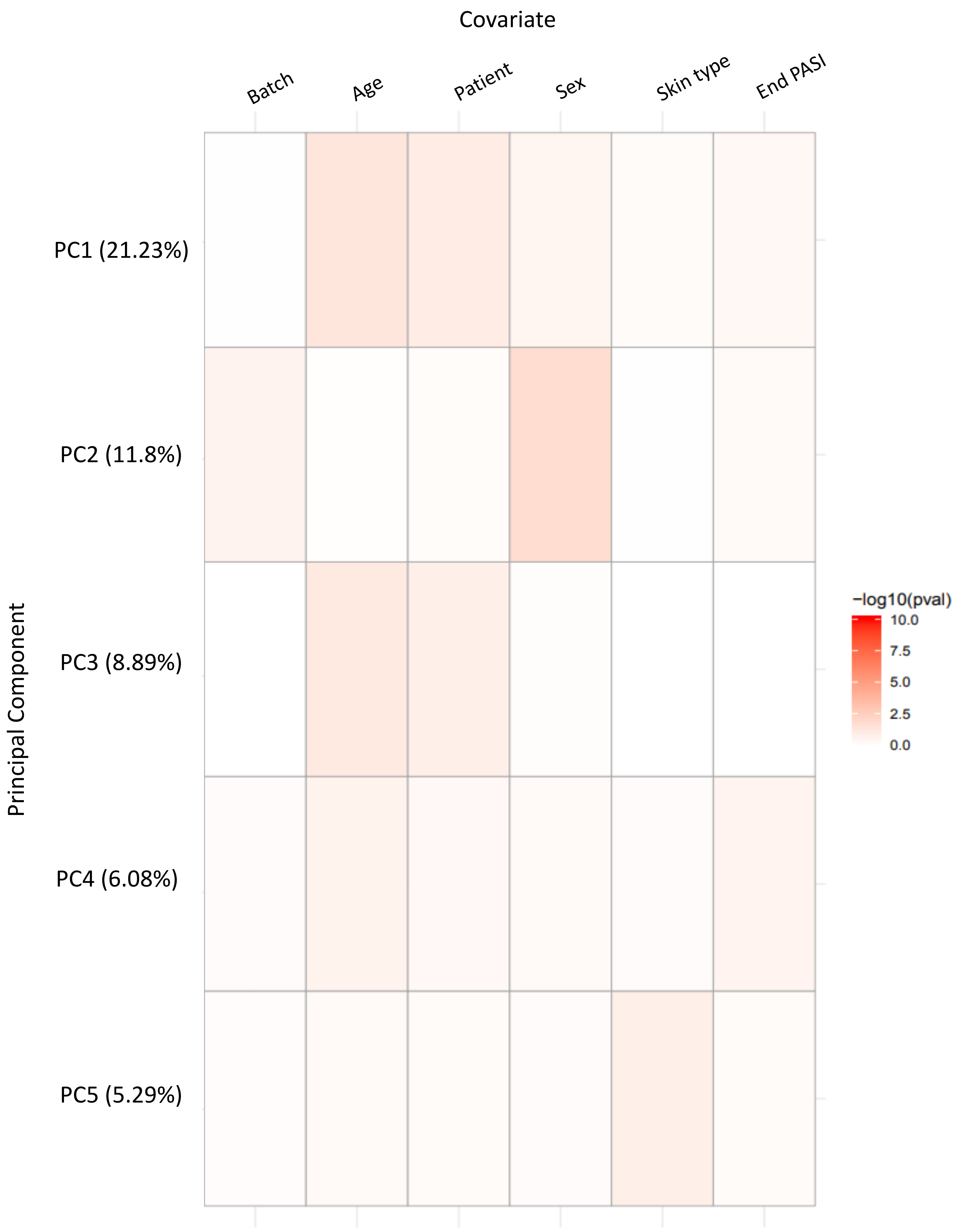

Supplementary figure 1. Principle component analysis (PCA) for patient demographics from historically obtained samples. PCA analysis was performed using the R package ggplot2. Each column represents a different patient demographic whilst each row represents the top 5 principle components for the microarray dataset accounting for a total variance equal to 53.29%.

Supplementary Figure 2

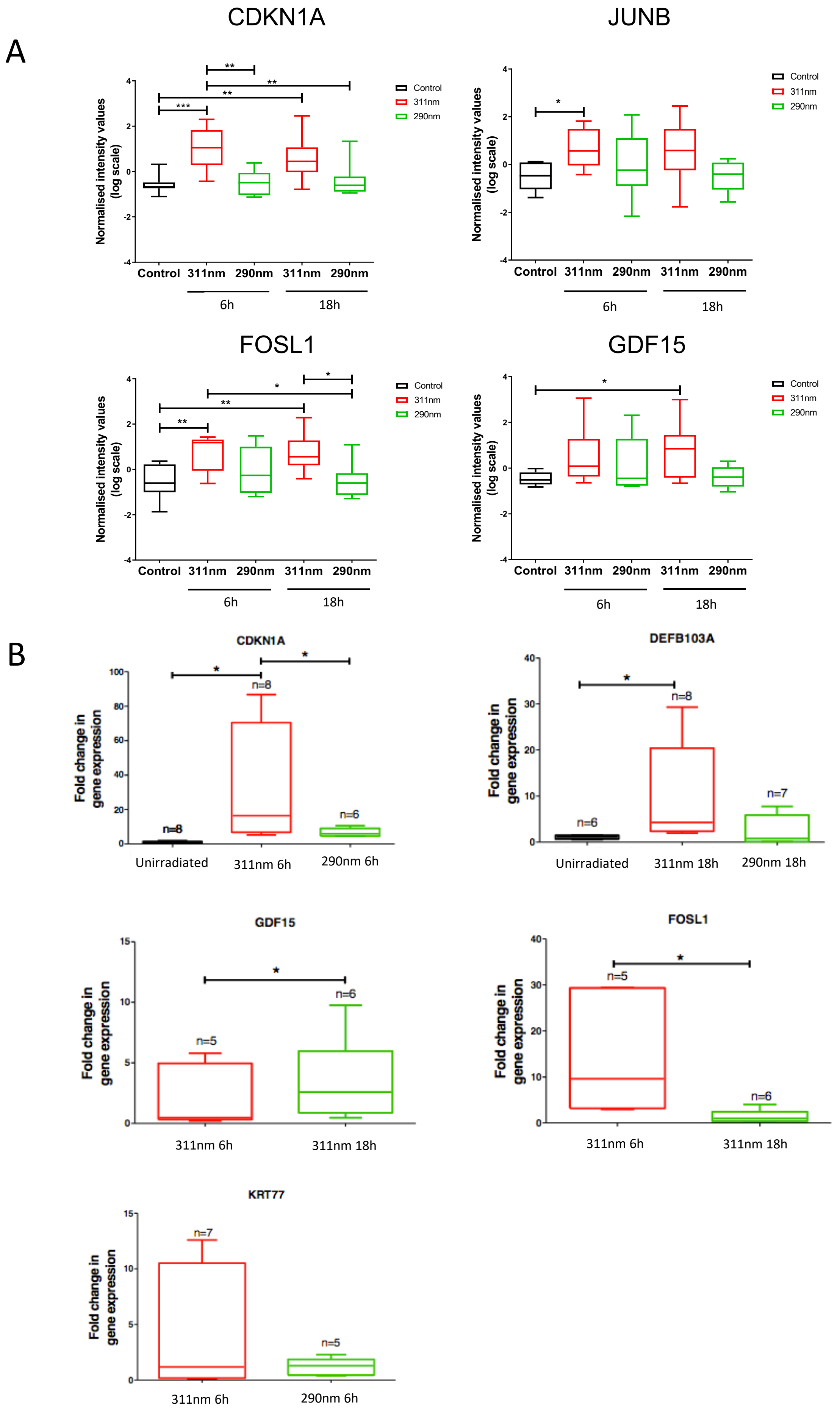

Supplementary figure 2. Expression fold change of genes in response to 311nm UVB and 290nm UVB at 6h and 18h post irradiation, compared to un-irradiated psoriasis.

A) Normalised intensity values are shown for CDNK1A (p21), FOSL1, GDF15 and JUNB. Median and inter-quartile range shown, with whiskers indicating minimum and maximum values. Graphs shows normalised values centred around zero. Red plots represent irradiation with 311nm UVB and green plots represent irradiation with 290nm UVB. *Significant difference shown ($p<0.05$) Mann Whitney-U test. B) PCR results for 2 differentially regulated genes within psoriatic epidermis. Up-regulation of CDKN1A at 6h post 311nm UVB and DEFB103A at 18h post 311nm UVB respectively compared to un-irradiated or 290nm-irradiated psoriasis. Median and IQR shown with whiskers indicating maximum and minimum values. * indicates significance at $p<0.05$ using a Mann Whitney-U test.

Supplementary Figure 3A

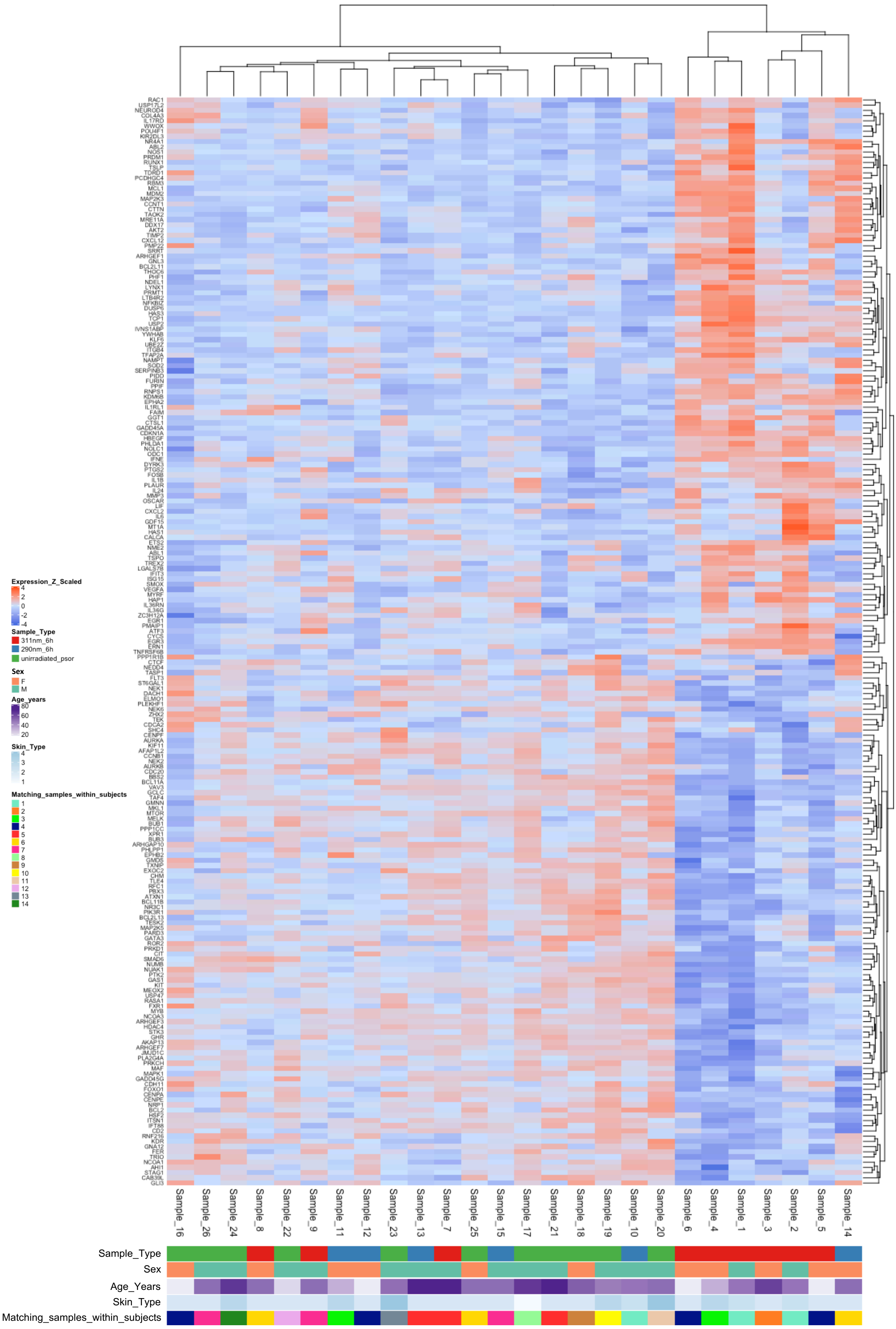

Supplementary figure 3. Identification of differentially expressed genes associated with apoptosis for patient biopsies irradiated with 311nm, 290nm or un-irradiated controls at 6h (A) and 18h (B). Heatmaps of normalised and batch corrected microarray gene expression data for DEGs associated with apoptosis and expressed in response to 311nm, 290nm UVB and un-irradiated lesional control at 6h (A) and 18h (B). Data was scaled prior to creating heatmaps (continuous variables placed on a unit scale (Z-scores)). Columns represent different sample types (see key) and rows represent apoptotic DEGs as labelled. Patient demographics are shown to demonstrate the relationship between these variables and apoptotic DEG expression. Sample annotations are shown at the bottom of the heatmap accordance with the key. Genes are clustered by an R pre-defined hierarchical clustering method. Unirradiated controls (Sample_16 – Sample_26) are present in both 4h and 18h heatmaps the remaining samples are unique to each heatmap.

Supplementary Figure 3B

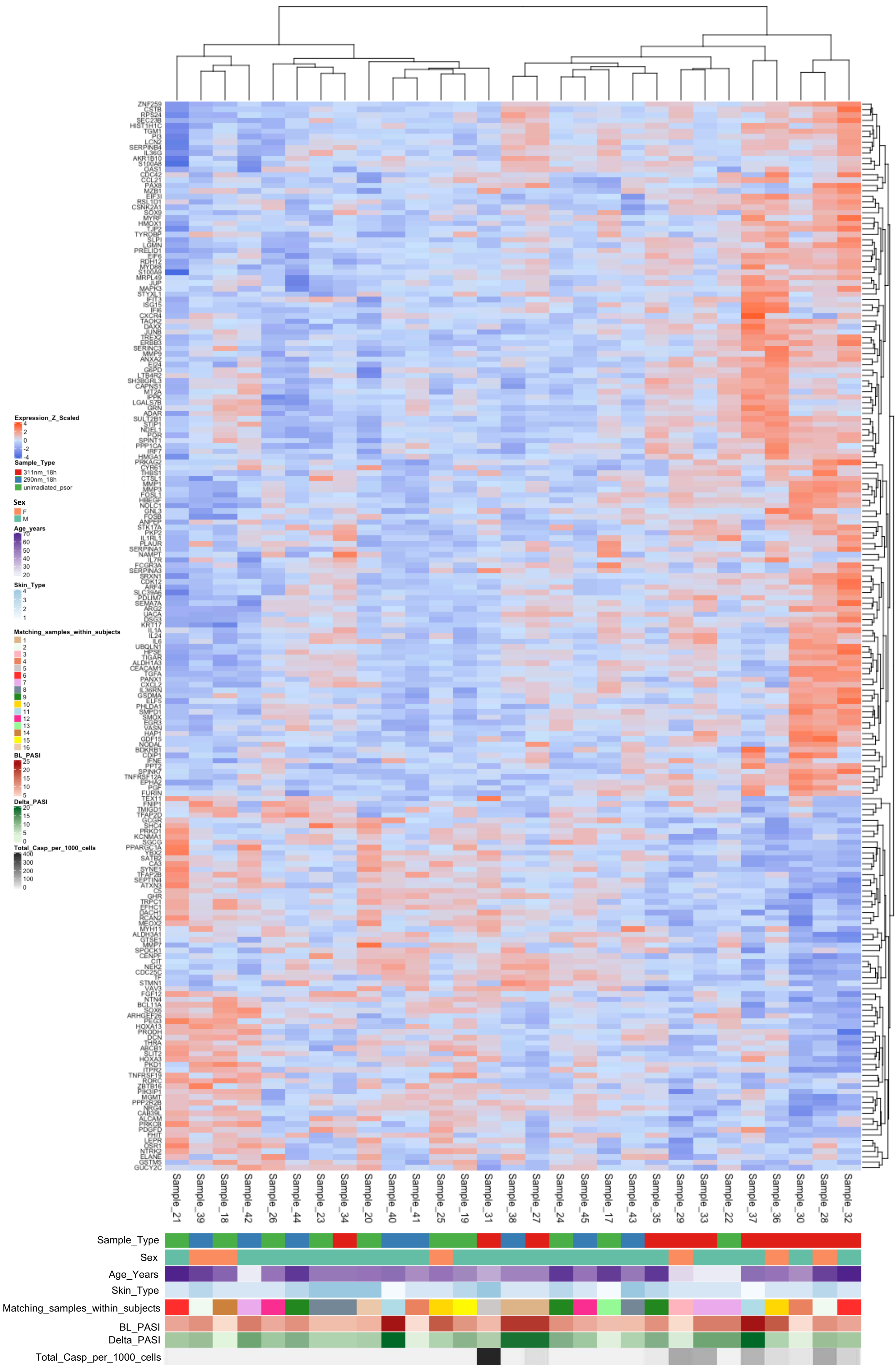

Supplementary Figure 4

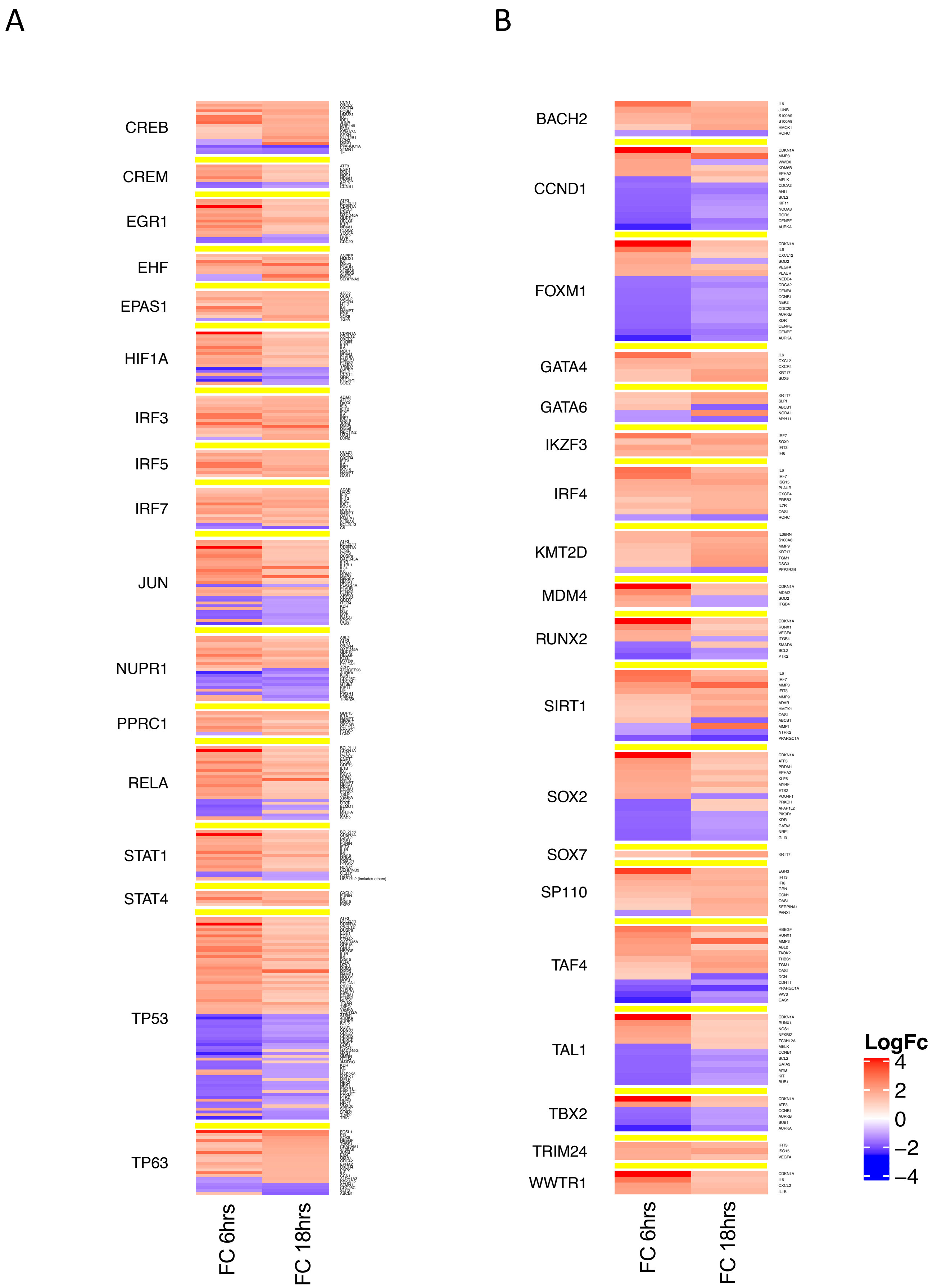

Supplementary figure 4. Identification of top upstream regulators at 6h and 18h post 311nm NB-UVB and associated change in gene expression of apoptotic DEGs at both time points. DEG expression of apoptotic genes (as demonstrated by the gene list on the right) based on the analysis of a combined total of 1510 DEGs. (A) demonstrates the top positive upstream regulators at 6h and 18h and (B) demonstrates the top negative upstream regulators at 6h and 18h. Yellow lines act to segment gene lists according to upstream regulators. Standardised gene expression levels shown as; red: high expression; blue: low expression.

Supplementary Figure 5

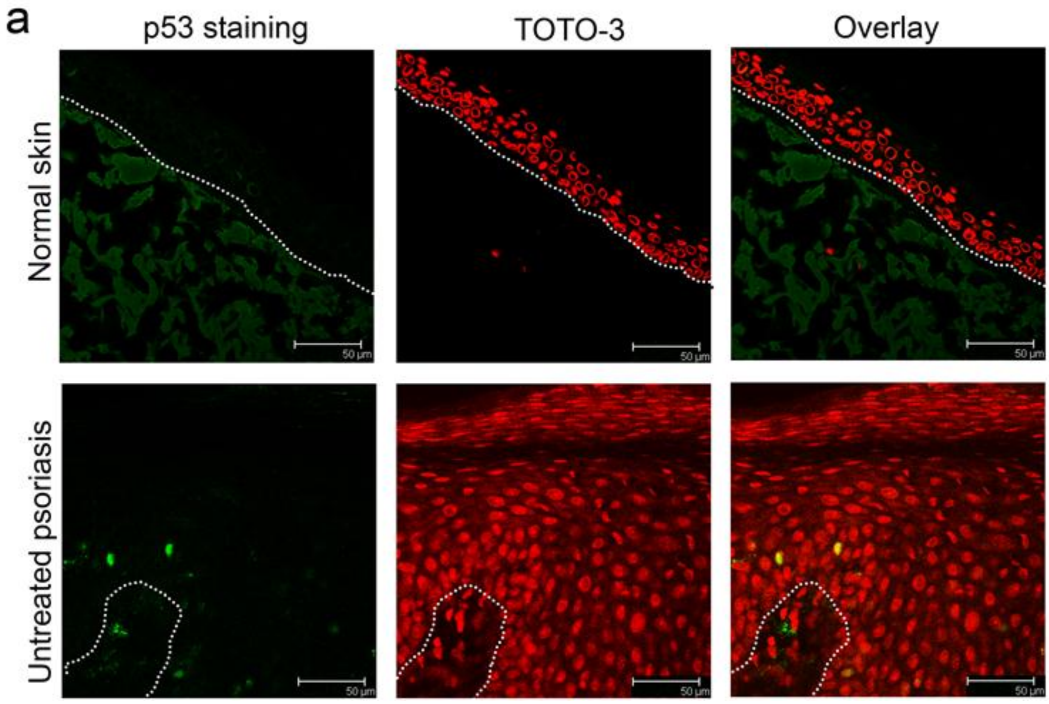

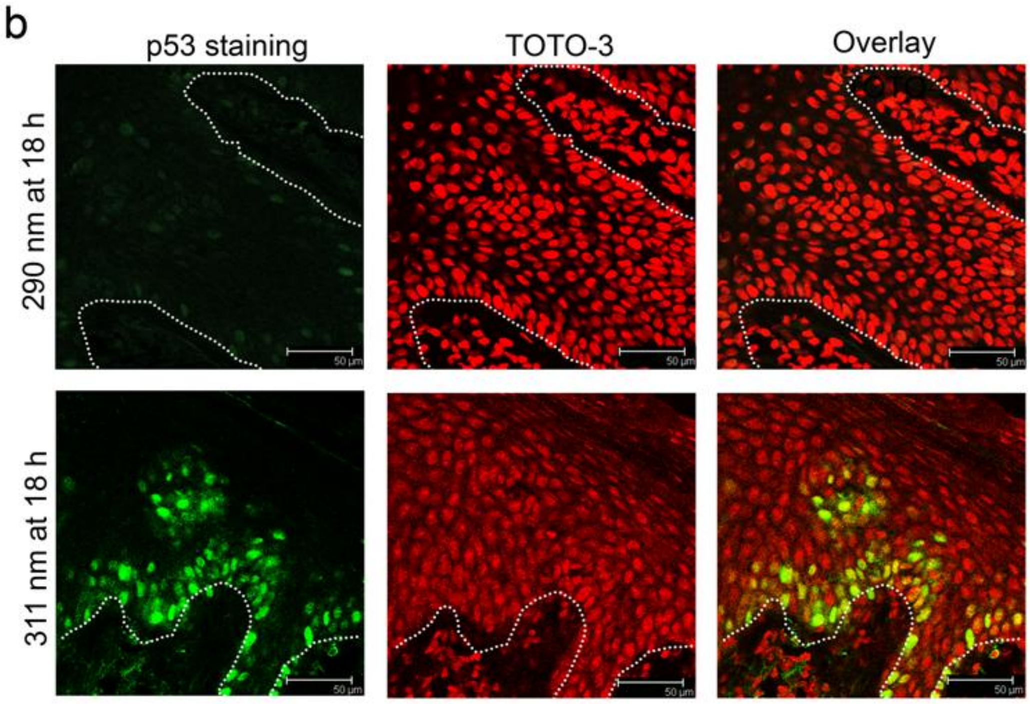

Supplementary figure 5. 311nm NB-UVB upregulates expression of nuclear p53 in human epidermis compared to 290nm and un-irradiated control. Immunostaining was performed using a p53 antibody (green fluorescent signal – anti-mouse rabbit Alexa Flour 488nm) and Toto-3 nuclear stain (red fluorescent signal – 642nm). (A) p53 positive cells were detected in normal skin and untreated psoriasis. (B) Low numbers of p53 positive cells 18h after irradiation of lesional psoriasis with 290nm UVB. Substantial increase in p53 nuclear staining and numbers of positive cells was observed in skin 18h after irradiation of lesional psoriasis with 311nm UVB (18h). Images are representative of 3+ donors. White dotted line denotes epidermal-dermal junction. All scale bars are 50µm.

Supplementary Figure 6

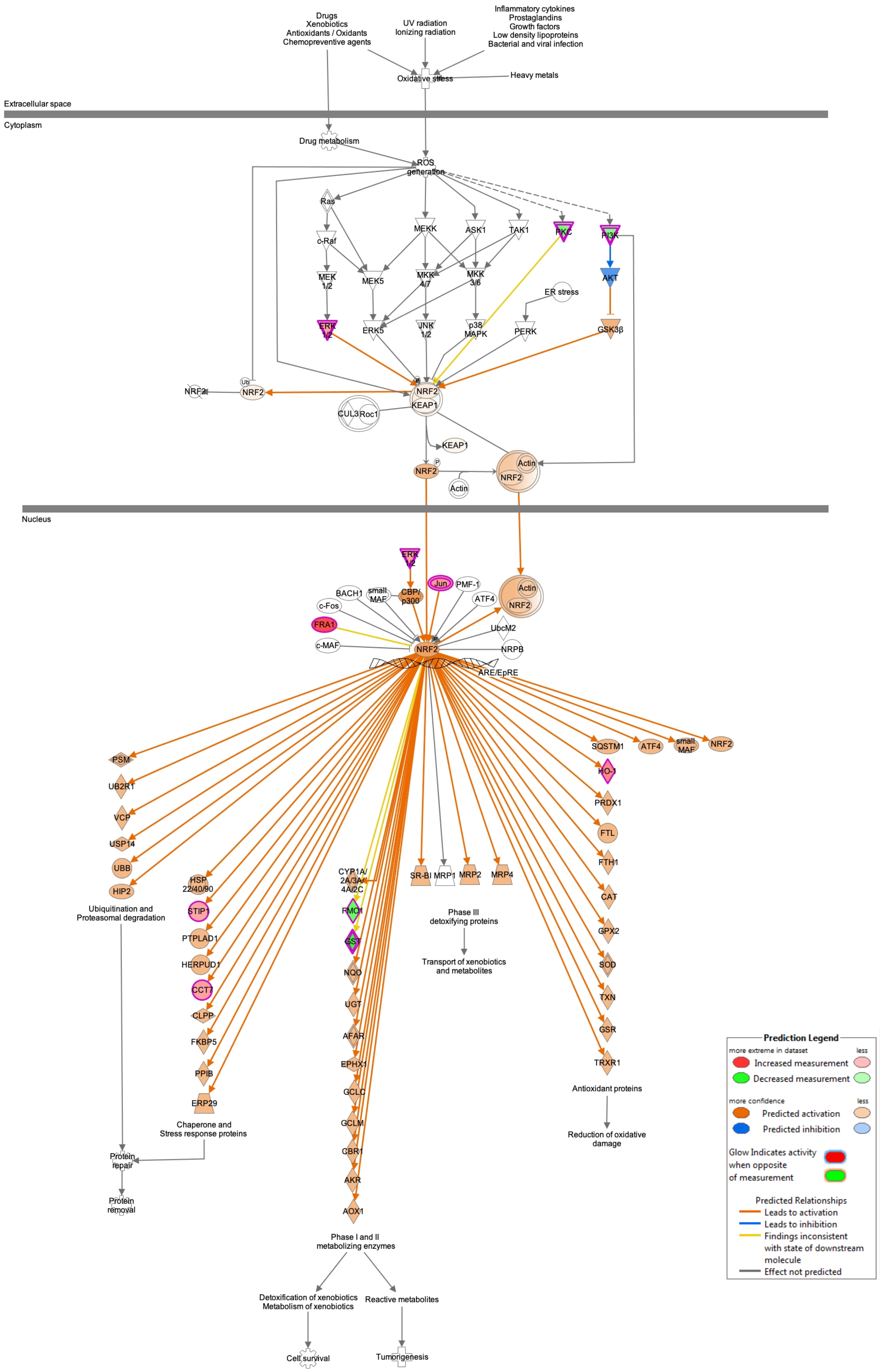

Supplementary figure 6. Enriched canonical pathways of the DEGs associated with the NRF2-mediated oxidative stress response signalling pathway identified by IPA, showing gene expression and predicted relationships between top regulated 311nm DEGs at 18h post irradiation. Signalling pathway upregulation based on the analysis of 795 DEGs respectively. Blue lines between DEGs represent predicted inhibition between genes and orange represent predicted activation between DEGs based on our transcriptomic data. Red symbols denote increased gene expression and green gene symbols represent downregulation. Each symbol shape represents a different molecule type.

Supplementary Figure 7

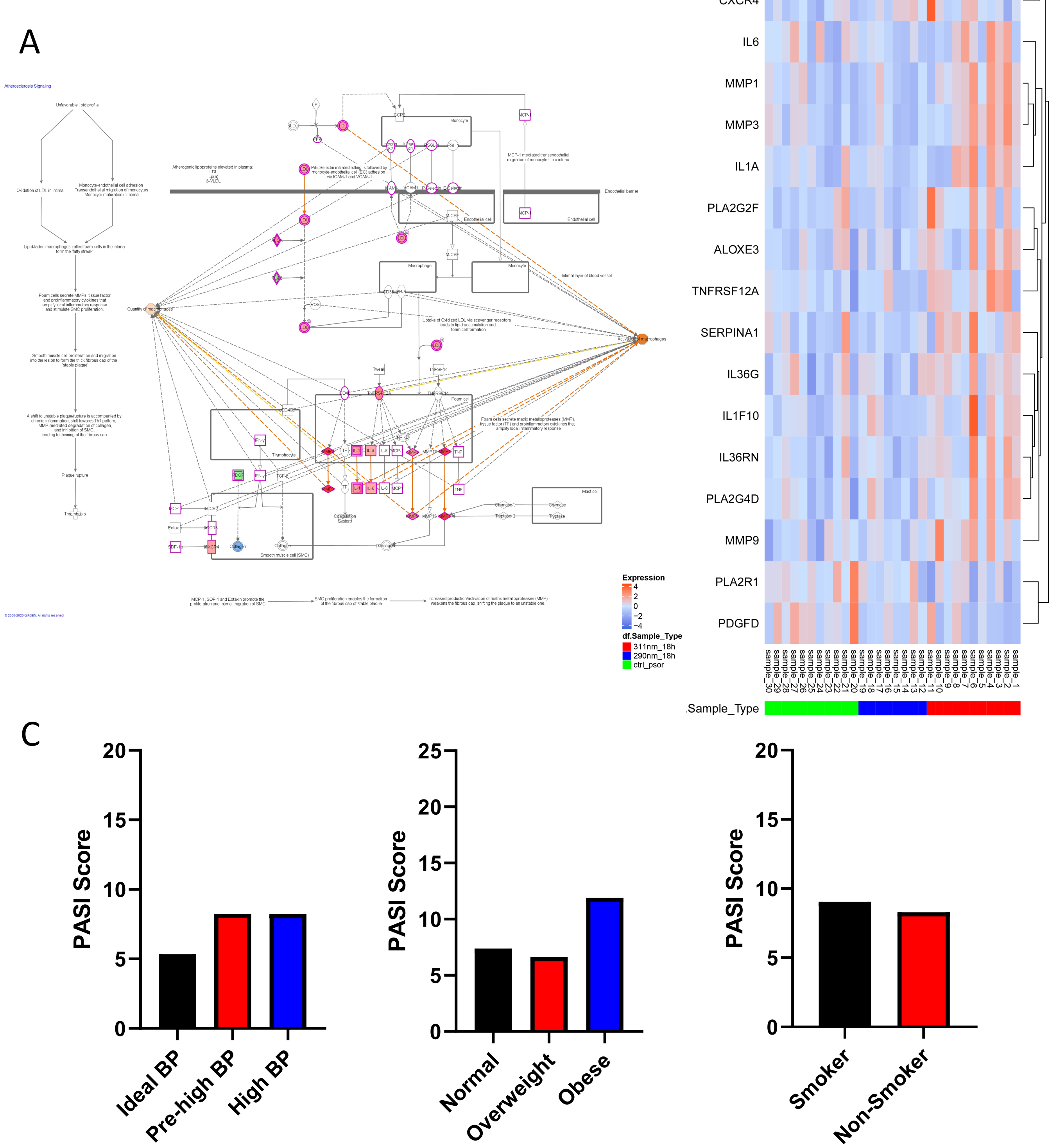

Supplementary figure 7. Enriched canonical pathways for the top ranked canonical pathway, atherosclerotic signalling at 18h post 311nm NV-UVB irradiation. DEGs associated with atherosclerosis when irradiated with either 311nm, 290nm and un-irradiated lesional controls are shown in the heatmap. (A) DEG expression and molecular relationships between DEGs predicted by using IPA molecular activity predictor (MAP) using our transcriptomic data of 795 DEGs (18h). DEGs associated with activation of macrophages and quantity of macrophages was applied to the atherosclerosis signalling pathway to identify any interconnectivity between atherosclerotic DEGs and these two processes. Blue lines between DEGs represent predicted inhibition between genes and orange represent predicted activation between DEGs based on our transcriptomic data. Red symbols denote increased gene expression and green gene symbols represent downregulation. Each symbol shape represents a different molecule type. (B) Heatmap shows normalised and batch corrected microarray gene expression data for DEGs associated with atherosclerosis expressed in response to 311nm, 290nm UVB and un-irradiated lesional controls. Column represent different sample types (see key) and rows represent DEGs as labelled (genes were scaled prior to analysis and were clustered by an R pre-define hierarchical clustering method. (C) Changes in PASI score from baseline to week 8 are shown using a one-way ANOVA this suggested no significance correlation between blood pressure, BMI or smoker status with PASI.

Supplementary Figure 8

A

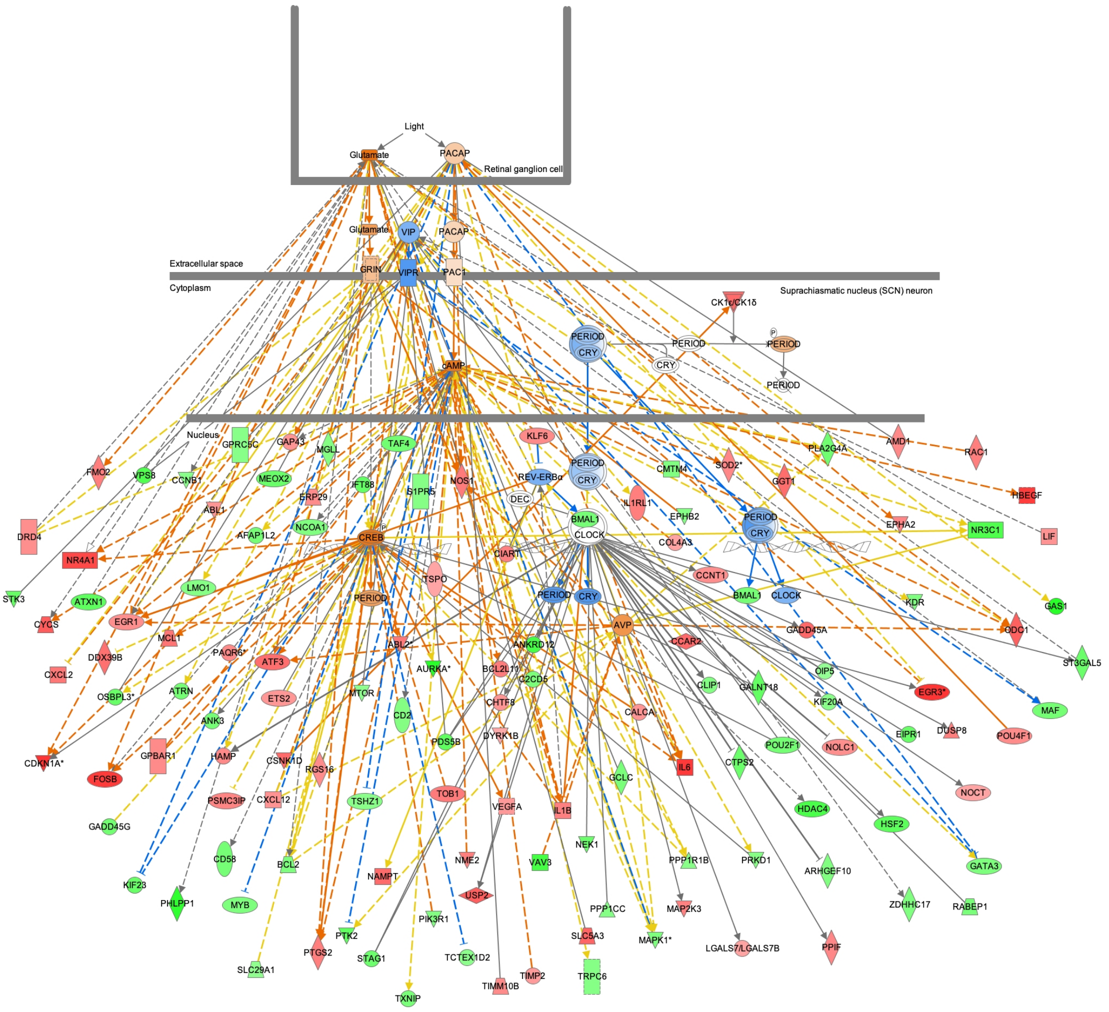

B

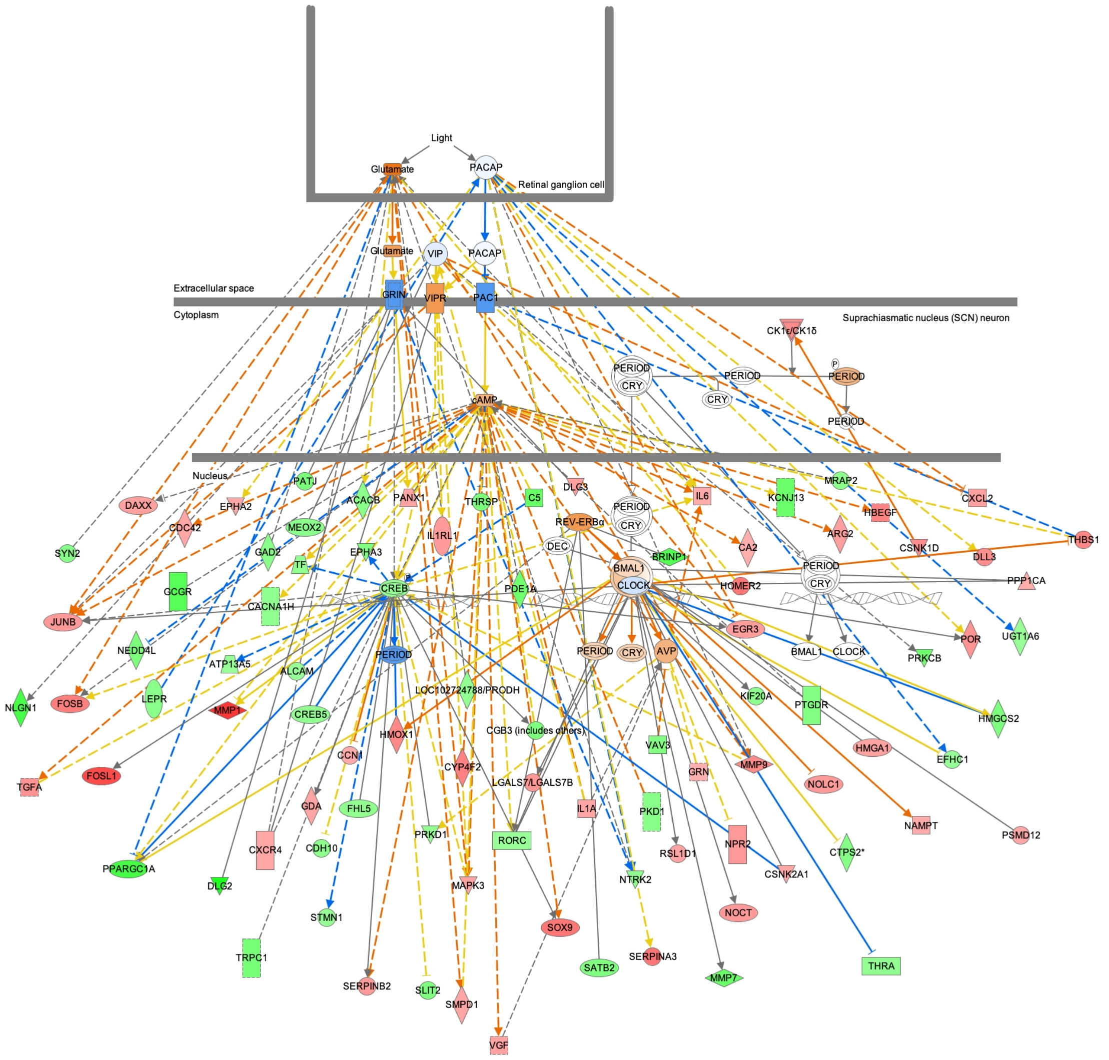

Supplementary figure 8. Enriched canonical pathways of the differentially expressed genes associated with transcriptional upstream regulators; CREM and CREB1 at 6h (A) and CREB1 18h (B) identified by IPA. Pathways show gene expression and predicted relationships between DEGs regulated within the circadian rhythm canonical pathway (retinal) in response to 311nm UVB. Signalling pathway upregulation based on the analysis of (A) 755 DEGs and (B) 795 DEGs respectively. Blue lines between DEGs represent predicted inhibition between genes and orange represent predicted activation between DEGs based on our transcriptomic data. Red symbols denote increased gene expression and green gene symbols represent downregulation. Each symbol represents a different molecule type.

Supplementary Figure 9

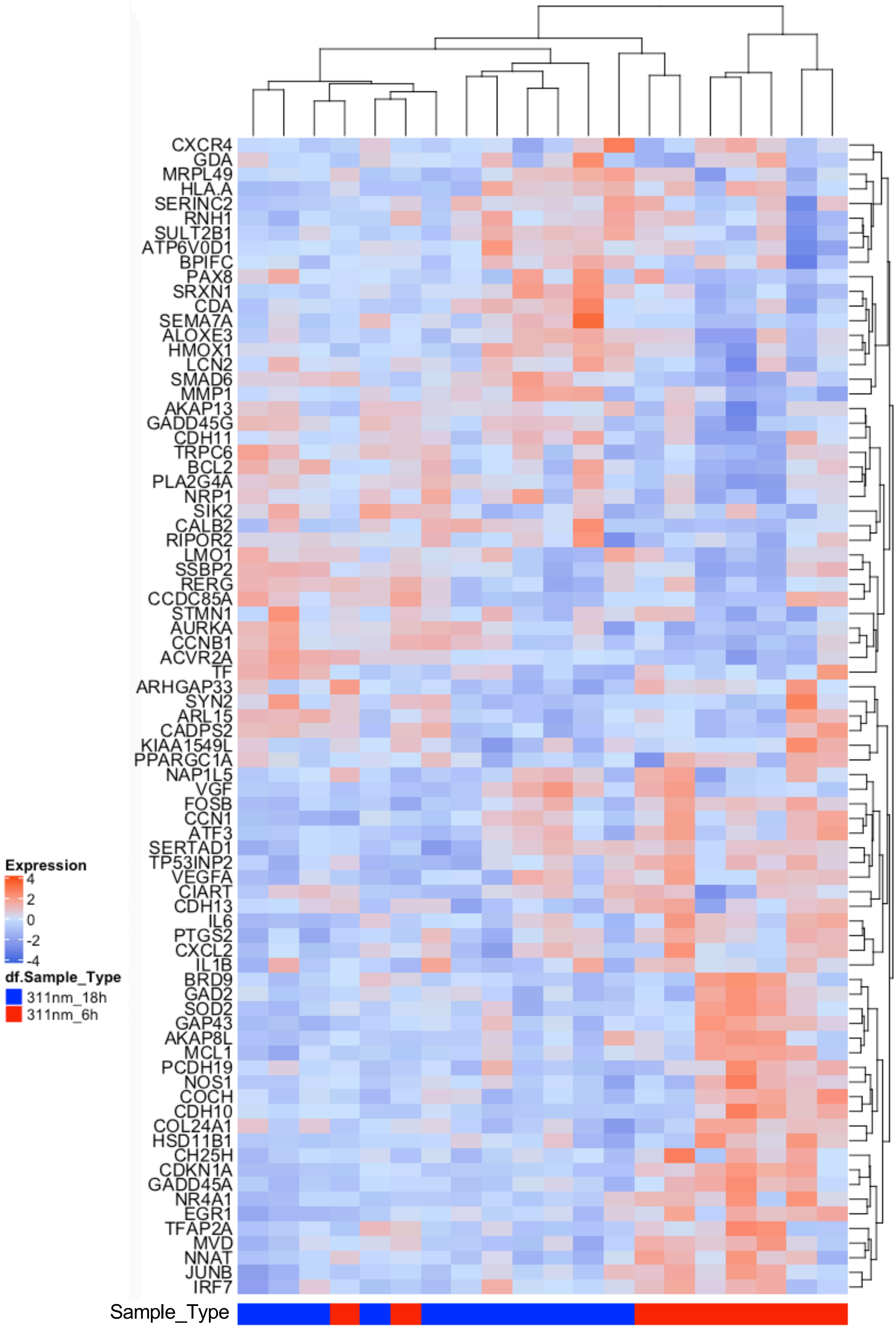

Supplementary figure 9. Identification of differentially expressed genes regulated by CREB1 for patient biopsies irradiated with 311nm UVB at 6h and 18h. Heatmaps of normalised and batch corrected microarray gene expression data for DEGs regulated by the top upregulated transcriptional regulator; CREB1 in lesional skin irradiated with 311nm UVB. Data was scaled prior to creating heatmaps (continuous variables placed on a unit scale (Z-scores)). Columns represent different sample types (see key) and rows represent CREB1 regulated DEGs as labelled. Genes are clustered by an R pre-defined hierarchical clustering method.

Supplementary Figure 10

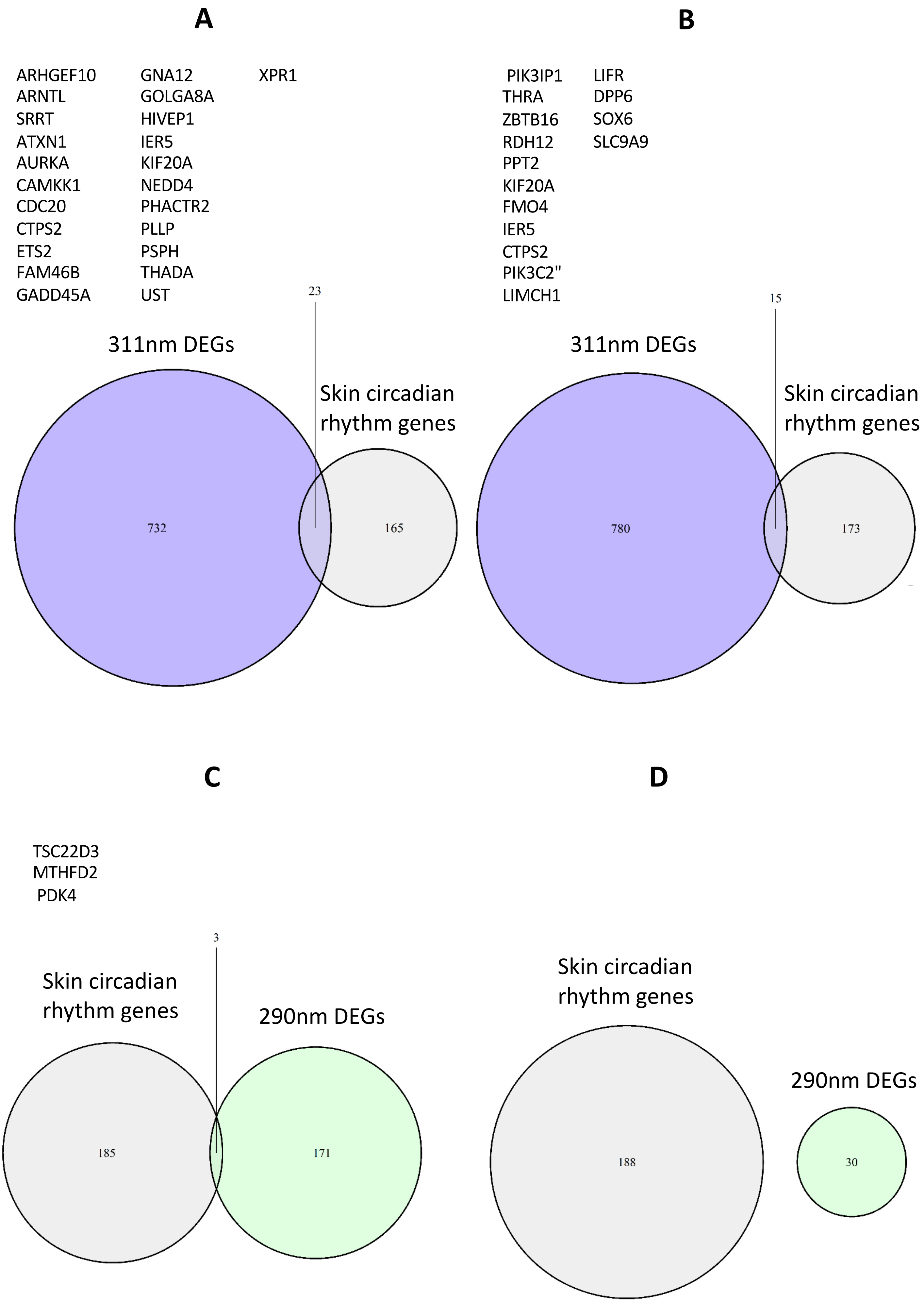

Supplementary figure 10: Overlap of skin circadian rhythm genes defined by Wu et al (2018, PNAS) and 311nm DEGs at 4 hours (A) and 18 hours (B), and 290nm DEGs at 4 hours (C) and 18 hours (D).

Supplementary table 1

See uploaded file entitled –
311nm_DEGs_at_6h_and_18h.xlsx

Supplementary table 2

A

ID	Genes in dataset	Pro-Apop	Anti-Apop	Our_Data
ILMN_1694757	CTSL	x		↑
ILMN_1790228	FURIN	x		↑
ILMN_1790659	GNL3	x		↑
ILMN_2097997	IFNE	x		↑
ILMN_2158713	IL36G	x		↑
ILMN_1804901	IL36RN	x		↑
ILMN_2064860	LGALS7/LGALS7B	x		↑
ILMN_1784459	MMP3	x		↑
ILMN_1811437	MYRF	x		↑
ILMN_2397199	NDEL1	x		↑
ILMN_2184640	NOLC1	x		↑
ILMN_1687978	PHLDA1	x		↑
ILMN_1755741	DACH1		x	↓
ILMN_1775814	GHR		x	↓
ILMN_2051373	NEK2		x	↓

B

ID	Genes in dataset	Pro-Apop	Anti-Apop	Our_Data
ILMN_1659800	BCL11A	x		↓
ILMN_1664516	CENPF	x		↓
ILMN_1695579	CIT	x		↓
ILMN_1807050	SHC4	x		↓
ILMN_2399463	VAV3	x		↓
ILMN_1682636	CXCL2		x	↑
ILMN_1701789	IFIT3		x	↑
ILMN_2054019	ISG15		x	↑
ILMN_1775380	SMOX		x	↑
ILMN_1791792	TIGAR		x	↑
ILMN_1680892	TREX2		x	↑

Supplementary table 2. – Identification of pro- and anti-apoptotic genes and their direction of change at 6h and 18h. DEGs associated with apoptosis were identified at both 6h and 18h post 311nm NB-UVB. DEGs shown were regulated at both 6h and 18h. Table A demonstrates DEG expression patterns which contribute to a pro-apoptotic environment whereas table B demonstrates DEG expression patterns which contribute towards and anti-apoptotic environment. Whether they are pro or anti apoptotic DEGs is shown by an X in the relevant column. The up and down arrows indicate the direction of gene expression within our dataset. 15 DEGs were regulated in a pro-apoptotic manner at both timepoints and similarly, 11 DEGs contributed towards an anti-apoptotic environment.

Supplementary table 3

Upstream regulator	Time point	Z score	Overlap P-Value
NUPR1	6h	3.683	2.99E-07
STAT1	6h	3.428	9.63E-03
RELA	6h	3.249	1.23E-04
EGR1	6h	3.080	1.63E-04
HIF1A	6h	2.976	8.02E-03
IRF7	6h	2.786	2.59E-02
JUN	6h	2.663	4.87E-07
PPRC1	6h	2.611	9.11E-04
CREM	6h	2.550	4.94E-03
TP53	6h	2.549	2.23E-08
CREB1	18h	3.837	3.55E-06
IRF3	18h	3.490	6.73E-05
NUPR1	18h	3.441	3.73E-01
IRF7	18h	3.010	8.36E-04
PPRC1	18h	2.910	5.44E-08
STAT4	18h	2.907	8.78E-02
EPAS1	18h	2.804	8.35E-02
IRF5	18h	2.711	2.08E-04
EHF	18h	2.598	7.31E-14
TP63	18h	2.564	2.31E-04

Supplementary table 3. Top upstream transcriptional regulators identified by IPA when ranked according to Z score for 6h and 18h post 311nm NB-UVB. Transcriptional regulators shown are ranked according to Z score at both time-points. Table also shows the overlapping p-values for each of the transcriptional DEGs. The top half of the table (halved by the bold black line) show DEGs differentially regulated at 6h and the lower half of the table shows DEGs regulated at 18h.
